# Contribution of artificial intelligence research to the development of inclusive applications for people with disabilities: A scoping review (2015–2025)

**DOI:** 10.1371/journal.pdig.0001749

**Published:** 2026-10-15

**Authors:** Soutongnoma Safiata Kaboré, Azadeh Bayani, Léandre Parfait Epoh Ewane, Davllyn Santos Oliveira dos Anjos, Jean Noel Nikiéma, Muriel Mac-Seing

**Affiliations:** 1 École d’optométrie, Université de Montréal, Montréal, Québec, Canada; 2 Centre de Recherche Interdisciplinaire en Réadaptation du Montréal métropolitain, Montréal, Québec, Canada; 3 Centre de recherche en santé publique, Montréal, Québec, Canada; 4 École de santé publique, Université de Montréal, Montréal, Québec, Canada; Khomein University of Medical Sciences, IRAN, ISLAMIC REPUBLIC OF

## Abstract

Artificial intelligence (AI) is rapidly transforming healthcare. Yet people with disabilities (PWD), representing 16% of the global population, remain disproportionately excluded from these advances. This exclusion is driven by algorithmic bias, non-representative datasets, and limited involvement of PWD in the design, development, and evaluation of AI applications. This scoping review examines the extent to which AI research in health contributes to the development of inclusive technologies for PWD, focusing on equity considerations. It identifies current trends, methodological gaps, and opportunities for socially responsive innovation. We conducted a systematic search across MEDLINE, Embase, Web of Science, and CINAHL for the 2015–2025 period, following Arksey and O’Malley’s five-stage scoping review methodology and the PRISMA-ScR guidelines. Data were extracted and synthesized thematically using the Population-Concept-Context approach. Of 4,233 references retrieved, 93 studies were included. Most studies originated from high-income countries, particularly in North America (45.2%) and Europe (26.9%). AI applications addressed motor, sensory, cognitive, communication-related, and psychosocial disabilities, with emphasis on prediction/diagnosis, assistive technologies, and personalized care. While about 60% of studies referenced equity, only 12 (12.9%) implemented concrete measures (e.g., adapting tools for vulnerable groups or ensuring sample diversity), and only six (6.5%) actively involved PWD through co-design or user validation. AI holds significant potential to enhance autonomy, access, and quality of life for PWD. Realizing this promise will require moving beyond technical optimization to embrace inclusive design, participatory methods, and equity-driven frameworks aligned with responsible AI development and use.

## Author summary

People with disabilities (PWD) are frequently underrepresented in the design, development, implementation, and evaluation of artificial intelligence (AI) systems, which can limit the accessibility, usability, and equity of these technologies, and result failing to address their diverse needs and lived experiences. It is therefore essential to evaluate the extent to which PWD are meaningfully involved across all stages of AI development and research. In this scoping review, we examined their roles from multiple perspectives. Our review explored how healthcare AI research contributes to the development of inclusive and accessible technologies for PWD. We identified studies addressing a wide range of disabilities and AI applications, including predictive models, assistive technologies, early screening tools, and decision-support systems. Overall, our findings highlight the growing recognition of inclusive AI development while demonstrating that meaningful participation, accessibility, transparency, and context-sensitive implementation remain inconsistently addressed. Based on these findings, we identify key gaps in the current literature and provide recommendations to guide the development of more equitable, accessible, and person-centered AI systems for people with disabilities.

## Introduction

Artificial intelligence (AI) has evolved from a research curiosity to a transformative force across sectors such as healthcare, transportation, finance, education, and public services [1,2]. Since 2015, advances in machine learning architectures, cloud-based computing, and sensor technologies have enabled AI systems to perform tasks once reserved for human expertise, such as diagnosing diseases from medical images or personalizing rehabilitation programs [3]. In parallel, normative frameworks like the Montréal Declaration for Responsible AI [4] and the United Nations Convention on the Rights of Persons with Disabilities [5] advocate for technologies that are inclusive, equitable, and socially sustainable. With people with disabilities (PWD) representing approximately 16% of the global population [6], the ethical imperative is clear: AI must be designed to empower rather than marginalize.

To date, most scholarly and industry-driven discussions on AI ethics focus on algorithmic bias related to race, gender, or sexual orientation, despite a call for universal design for all [7,8]. However, disability remains underexplored, often treated as a statistical anomaly rather than a user group whose lived experiences should shape system requirements [9]. Empirical consequences are evident: large training datasets frequently omit disability-related variables (e.g., assistive device usage, functional scores), compromising model generalizability for PWD [10,11]. Designers cite the heterogeneity of PWD as a barrier, leading to their exclusion in methodologies in early design phases and user testing [7,9,10]. Resulting AI applications (diagnostic classifiers, predictive risk tools, or assistive interfaces) are seldom evaluated for accessibility, cultural relevance, or intersectional equity [7,11]. These gaps reflect a structural reproduction of ableist norms, risking the amplification of existing health and social inequities, further marginalizing PWD [12]. Taken together, these limitations reveal a critical blind spot: the field lacks a systematic evidence base showing where disability is meaningfully included, or omitted, across AI health-related research. Systematically mapping patterns within the peer-reviewed literature can help researchers, developers, and decision-makers identify gaps in disability-inclusive AI research. However, policy development also requires evidence from grey literature, community-led initiatives, participatory research, and implementation experiences that extend beyond the scope of this review. Over the 2015–2025 period, this scoping review addresses this knowledge gap by systematically mapping: 1) the spectrum of AI techniques and application domains deployed for PWD in the field of health, identifying methodologies and areas for innovation; 2) the distribution of disability categories, geographic contexts, and study designs, revealing structural biases that limit external validity; and 3) the operationalization of equity, diversity, and inclusion principles, including examples of bias mitigation, culturally adapted datasets, and participatory co-design. This study is timely because disability inclusion, accessibility, and equity are increasingly recognized as essential components of responsible AI and contribute directly to global efforts toward reducing inequalities and promoting inclusive health systems.

## Materials and methods

This scoping review is part of a broader research project which explores the intersections between AI, disability, and equity issues. The scoping review protocol has been registered on the Open Science Framework platform [13]. It followed the five-step methodology proposed by Arksey and O’Malley [14] 1) identifying the research question; 2) searching for relevant studies; 3) selecting studies; 4) extracting data; 5) synthesizing and presenting results. Methodological recommendations from Levac et al. were also integrated to enhance the clarity of objectives and the rigour of the process [15]. Additionally, the review adhered to the PRISMA-ScR (Preferred Reporting Items for Systematic Reviews and Meta-Analyses extension for Scoping Reviews) guidelines [16]. Specifically, we will use the Population-Concept-Context (PCC) approach recommended by the Joanna Briggs Institute for scoping reviews [17]. In this study, the “Population” refers to PWD; the “Concepts” pertain to AI, equity, diversity, and inclusion; and the “Context” encompasses any AI applications related to the health sector. At the conceptual level, our scoping review is guided by the framework of intersectionality, which critically analyses the relationships between identities, equity, societal structures and technology. Intersectionality allows for a systematic exploration of how social identities, such as disability, gender, race, and other social markers, interact with societal power structures (such as ableism) to produce specific forms of exclusion in digital environments [17,18]. This approach is particularly relevant for understanding the combined effects of ableism, a structural power that discriminates against PWD, and other forms of discrimination in the design of AI algorithms and applications [19].

### Information sources and search strategy

The literature search strategy was developed in close collaboration with a public health librarian from the Université de Montréal to ensure the rigour, comprehensiveness, and alignment of the process with the review’s objectives. It was initially developed and tested in the Medline (Ovid) database, then adapted and applied to three additional databases: Embase, CINAHL, and Web of Science. Searches were conducted in both French and English, covering publications from January 2015 to May 2025 to reflect recent research and development advances in the healthcare field of AI applied to PWD. The search terms included keywords and MeSH terms, selected to capture dimensions related to AI, inclusion, and various types of disabilities (S1 Text).

#### Eligibility criteria.

Eligible studies included original quantitative and qualitative research, as well as peer-reviewed commentaries, that explore concepts related to AI and directly or indirectly address issues of equity, diversity, disability, or innovation in health-related research and development. Publications written in French or English and conducted between January 2015 and May 2025 were considered. Exclusion criteria included letters to the editor, editorials, perspectives, conference proceedings, opinion pieces, posters, and interviews. Additionally, any publication that could not be retrieved in full text or that was published in a language other than French or English was excluded.

### Study selection

Three reviewers (AB, LPEE, KSS) independently screened the search results according to the eligibility criteria in two stages: 1) title and abstract screening; 2) full-text review. Discrepancies were resolved through discussion with two other reviewers (DSOA, JNN and/or MMS), and the criteria definition was adjusted accordingly. A pilot test of title and abstract screening was conducted on the first 50 references. A flow diagram generated by the Covidence interface was produced to document the study selection process and is presented in the results section.

### Data management, extraction, and analysis

Results from the bibliographic databases were first imported into the Covidence application availed by the Université de Montréal, which automatically removed duplicates of references. Selected references were then transferred to Zotero (Version 5.0.96.3) for structured and centralized bibliographic management. Data extraction was performed using a standardized Excel spreadsheet to ensure systematic and consistent collection of information. Extracted elements included: bibliographic data (title, authors, year of publication), country/region where the study was conducted, study objectives, types of disabilities addressed, AI models used, and types of applications developed. Also noted were considerations related to equity, diversity, and inclusion, as well as implications for research, public policy, and professional practice. Equity, diversity, and inclusion were operationalized a priori using predefined indicators. These included participatory design or co-design approaches, involvement of people with disabilities in development or validation processes, representation of diverse populations, accessibility adaptations, culturally responsive implementation strategies, consideration of underrepresented groups, and explicit efforts to identify or mitigate algorithmic bias [20]. Data analysis was based on a descriptive thematic approach, allowing results to be grouped into coherent thematic categories. This method facilitates the identification of dominant trends, methodological gaps, and emerging opportunities in the integration of AI to serve PWD.

## Results

A total of 4,233 records were retrieved from four databases: Embase (n = 2,306), Medline (n = 1,354), Web of Science (n = 300), and CINAHL (n = 273). After removing 413 duplicates, 3,820 titles and abstracts were screened. Of these, 201 articles were selected for full-text review based on predefined inclusion and exclusion criteria. Following this process, 93 studies were included in the final stage of the review for analysis. The PRISMA flow diagram (Fig 1) summarizes each step of the selection. Among the 93 included studies, 57 (61.3%) studies employed quantitative methods, focusing on clinical, behavioural, or technological data, while 16 (17.2%) studies used qualitative approaches, exploring user experiences and perceptions through interviews, observations, or content analysis or a reflection-based approach. These results provide a descriptive overview of the methodological orientations in current research on disability and AI.

**Fig 1:**
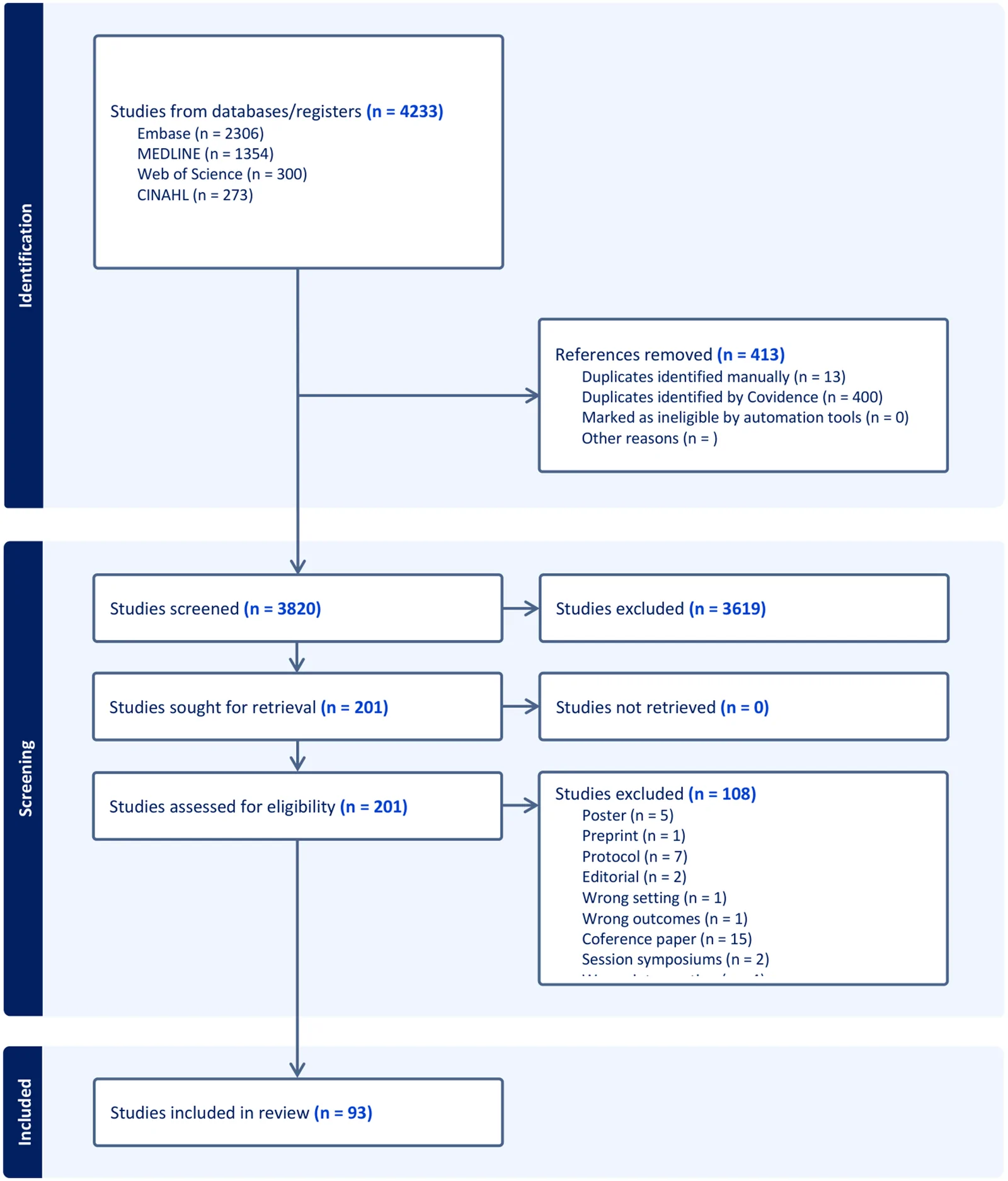
Flow chart.

### Characteristics of included studies

The studies included in this review originate predominantly from North America (45.2%), followed by Europe (26.9%) and Asia (28.0%), with limited representation from Oceania (4.3%) and Africa (1.1%). Studies focused primarily on cognitive disabilities, such as Alzheimer’s disease, autism spectrum disorders, and intellectual disabilities. In addition, several studies addressed mobility-related conditions, communication disorders, and hearing disabilities, while visual disabilities are examined less frequently. In terms of technological approaches, the most common applications of artificial intelligence were predictive models, which were complemented by classification systems and physical assistive technologies such as brain-computer interfaces and robotic exoskeletons. More recent contributions also explored bias detection and analysis tools, reflecting the growing concern for fairness and equity in AI systems. Overall, these technologies aim to enhance diagnosis, support rehabilitation, enable personalized care, and promote digital inclusion for persons with disabilities. The characteristics of the included literature have been summerized in (S2 Text).

### Geographic representation of studies on AI applied to disability: Mapping global scientific inequalities

The analysis of 93 peer-reviewed studies on AI applied to disability reveals a pronounced imbalance in global scientific production mostly produced in the Global North. As shown in Fig 2, among the included studies, 68 (73.1%) originate from high-income economies (HICs) and 25 (26.9%) from low- and middle-income economies (LMICs). Within HICs, the United States of America (USA) leads with 28 studies [21–48], followed by Canada [49–52], France [53–56], and the United Kingdom (The UK) [43,57–59]. Other HIC contributors include South Korea [60–62], Italy [63–65], the Netherlands [66–68], and Spain [69–71]; Singapore [72,73], and Australia [74,75]; as well as single-study contributions from Portugal [76], Belgium [77], Switzerland [78], Denmark [79], Norway [80], Germany [81], Japan [82], New Zealand [83], Romania [84], and Saudi Arabia [85]. Two multi-regional entries USA & Canada [86] and Europe [87] are also included and were counted under HICs given their composition. In LMICs, China leads with 13 studies [47,57,88–98], followed by Iran [99–101], India [102,103], and Mexico [104,105]. Single-study LMIC contributors include Turkey [106], Zambia [107], Fiji [108], Argentina [109], and Thailand [110].

**Fig 2:**
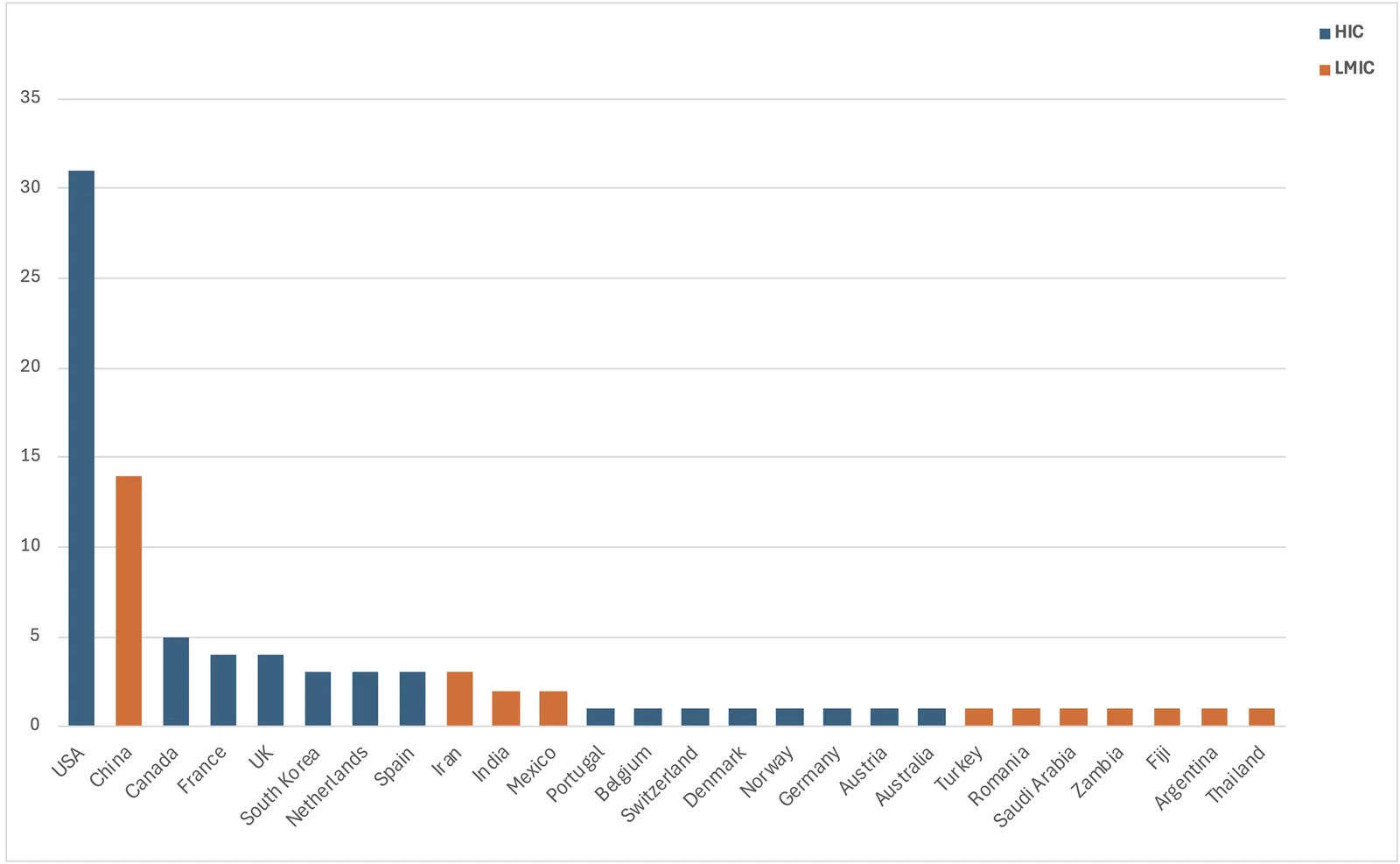
Geographic distribution of studies included in the review.

### Typology of disabilities targeted by AI applications in the sector of healthcare

The studies included in this review explored a wide range of disability situations, reflecting the growing interest in applying AI across healthcare. Disabilities have been defined broadly according to WHO (e.g., motor, sensory, communication and cognitive and mental disorders), along with emerging disability categories related to illnesses and functional impairments [6].

These categories were used as a pragmatic framework to facilitate the synthesis of the heterogeneous literature included in this scoping review.

#### Motor disabilities.

Mobility-related challenges were addressed in 28 studies (30.1%). These contributions applied AI to improve functional mobility, rehabilitation outcomes, and clinical decision-making across a wide spectrum of neurological and musculoskeletal conditions. Eight studies contributed on predictive modeling to estimate post-stroke motor recovery trajectories [26,27,62,72,75,83,105,110], seven studies focused on forecasting disability progression in multiple sclerosis (MS) or spinal disorders [23,24,37,51,67,77,80], and three studies on characterizing biomechanical patterns relevant to surgical planning or risk assessment [21,55,93]. A second group of studies focused on AI-enabled assistive technologies, including exoskeletons [53], robotic orthoses [72,105], bionic reconstruction algorithms [74], and brain-computer interfaces (BCIs) for motor intention detection and neuromodulation [26,62,105]. Complementary research advanced wearable-sensor gait analysis and movement classification to quantify functional arm or gait activity in real-world settings [27,75,76], while others introduced tongue-driven control systems enabling alternative interaction pathways for individuals with severe mobility limitations [108]. AI was also used to support administrative modeling of disability and risk stratification, notably through automated extraction of electronic health records [21], classification of functioning and participation levels in hospitalized patients [68], and development of disability algorithms leveraging large administrative datasets [51]. Additional studies explored the potential of AI to improve vocational rehabilitation outcomes [37], enhance inclusive motor-based educational settings such as adapted dance programs [91], and analyse the interaction between motor and communicative functions, including multimodal environments and auditory-motor integration [59,78].

#### Sensory disabilities.

Sensory disabilities particularly concerning vision and hearing were examined in nine studies (9.7%) of the reviewed literature. Across these contributions, AI was primarily applied to enhance screening, diagnostic accuracy, and functional interaction for individuals with sensory limitations. In the domain of visual disabilities, AI models were developed to automate the detection of diabetic retinopathy and related retinal conditions [73,107], while other studies proposed methods to analyse visual markers or integrate visual information into assistive tasks such as sign-language recognition [103]. Additional research explored how visual cues contribute to emotion recognition in populations with neurodevelopmental conditions [81]. Hearing-related studies leveraged AI to interpret audiograms with high accuracy [49], predict vestibular markers such as Subjective Visual Vertical (SVV) and ocular Vestibular Evoked Myogenic Potential (o-VEMP) [28], and estimate hearing impairment in occupational contexts [99]. Other contributions examined real-world auditory environments and the influence of soundscapes on social participation [41], or assessed diagnostic tools for conditions with auditory-processing dimensions such as autism spectrum disorders (ASD) [61].

#### Cognitive and mental disorders.

In this section, studies relate to Autism spectrum disorders (ASD), which was examined in six studies (6.5%) of the included literature [38,45,56,58,98,106]. These studies explored early detection, behavioural modeling, and communication support, leveraging a variety of AI-driven methodologies. Several studies focused on early screening and behavioural identification, notably through eye-tracking-based machine-learning systems designed to detect ASD-related visual attention patterns in young children [106]. Deep learning applied to acoustic voice features was used to classify ASD and differentiate it from heterogeneous control groups [56]. In clinical settings, biometric and wearable sensors enabled the prediction of imminent aggressive behaviour among autistic youth, demonstrating the feasibility of real-time behavioural risk assessment [38]. Multimodal studies also examined sensory integration, with work evaluating atypical modulation of peripersonal space (PPS) during social interactions, combining psychophysical measures, electrophysiology, and neural network modeling [58]. Additional research leveraged AI to identify predictors of language gains in minimally verbal autistic children, supporting more personalized intervention strategies [45]. Efforts in early diagnosis extended to machine-learning detection of neurodevelopmental features in very young children, emphasizing the importance of early-life biomarkers [98].

#### Cognitive, neurodegenerative, intellectual, and neurodevelopmental conditions.

Cognitive and neurodegenerative disorders were addressed in 49.5% of the studies (N = 46), making this the most extensively represented category in the literature. These studies covered a broad range of conditions, including Alzheimer’s disease, dementia, mild cognitive impairment (MCI), Down syndrome, intellectual disability, dyslexia, and other neuropsychiatric symptoms [22,29,34,35,44,47,48,63,66,70,79,82,85–90,92,94,95,102,104,111,112]. Alzheimer’s disease and related dementias represented the largest component of this category. Across this category, AI was primarily used to identify biomarkers, predict disease progression, and improve diagnostic accuracy, often through the integration of multimodal data such as neuroimaging (MRI, fMRI), electrophysiology (EEG, fNIRS), behavioural measures, clinical records, and administrative data [34,36,40,47,70,79,86,88,94,111]. Several studies developed AI-based systems for stress detection in individuals with intellectual disabilities [66] or conducted large-scale analyses of clinical narratives to identify predictors of cognitive decline and functional impairment [35,44,68]. EEG-based and multimodal emotion-recognition models were employed to monitor therapeutic responses and behavioural states in individuals with neurodevelopmental disorders, including Down syndrome and intellectual disability [82,102,104]. In parallel, natural language processing (NLP) techniques were used to characterize agitation, communication difficulties, and other behavioural manifestations associated with dementia and related disorders [35,42,44]. Finally, several predictive models focused on estimating depression risk among caregivers of individuals with dementia, highlighting the broader societal and policy relevance of AI-driven insights in the context of cognitive and neurodegenerative disorders [52,60].

#### Communication and psychosocial disabilities.

Communication disabilities were explored in seven studies (7.5%), primarily through AI applications targeting speech/language assessment and classification. Approaches included speech analysis for aphasia using connected-speech features [33], mobile/automated screening for speech-sound disorders in preschoolers [84], and language-feature modeling in Alzheimer’s disease with multilingual NLP descriptors [42]. Additional studies examined predictors of clinical identification of language disorders in young children [39], ASR-based strategies for dysarthria in Parkinson’s disease [65], eye-tracking/ML screening relevant to communicative behaviours in ASD [106], and social-context sensory integration affecting communicative space (peripersonal space) in autistic participants [58].

### Artificial intelligence applications: A functional mapping

The analysis of the 93 studies included in this review reveals a remarkable diversity in the use of AI in the health sector intersecting with disability. Far from being a mere technical tool, AI is deployed as a lever for clinical, social, and technological innovation, aiming to improve autonomy, quality of life (QoL), and access to care. The applications identified have been grouped into seven (7) major functional categories: 1) clinical and behavioural prediction; 2) automated classification; 3) early detection and screening; 4) clustering and unsupervised segmentation; 5) intelligent assistive technologies; 6) decision support systems; and 7) semantic and language analysis. In addition to these seven categories, a cross-cutting theme related to equity considerations emerged across several studies. The main AI categories and their corresponding subcategories are presented in Table 1. Emerging domains such as intelligent assistive technologies, early screening tools, clustering methods, semantic analysis, and even equity-oriented approaches demonstrate the capacity of AI to go beyond data analysis and become a driver of clinical, social, and ethical innovation. Yet, these developments remain fragile due to recurrent methodological issues (small sample sizes, heterogeneous data, lack of external validation). Fig 3 illustrates the functional mapping of AI applications.

**Table 1:** Classification of AI applications.

Main categories (percentage of studies)	Subcategories	Examples of application
**Clinical & behavioural prediction (72%)**	Mobility-related conditions	Prediction of mobility impairments
	Cognitive & neurodegenerative disorders	Prediction of dementia progression
	Hearing-related disorders	Hearing loss prediction
	Communication/developmental conditions	Autism-related prediction
**Automated classification (7.5%)**	Vision	Classification of visual impairments
	Physical function after stroke	Motor functional classification
	Cognitive performance improvement	Brain-computer interface optimization
**Early detection & screening (7.5%)**	Neurodegenerative conditions	Early Alzheimer’s detection
**Clustering & unsupervised segmentation (4.3%)**	Spinal deformity groups	Patient stratification
	Latent profile detection	Dementia cases in a longitudinal setting
**Intelligent assistive technologies (9.7%)**	Daily activity support	Mobility/ autonomy tools
	Real-time detection	Obstacle detection for autism
**Decision support systems (3.2%)**	Care pathway optimization	Clinical decision tools
	Predictive models	Predictions for personalised intervensions
**Semantic & language analysis (2.2%)**	Language impairment detection	NLP for Alzheimer’s detection
	Cognitive impairement detection	Vocal data detection

**Fig 3:**
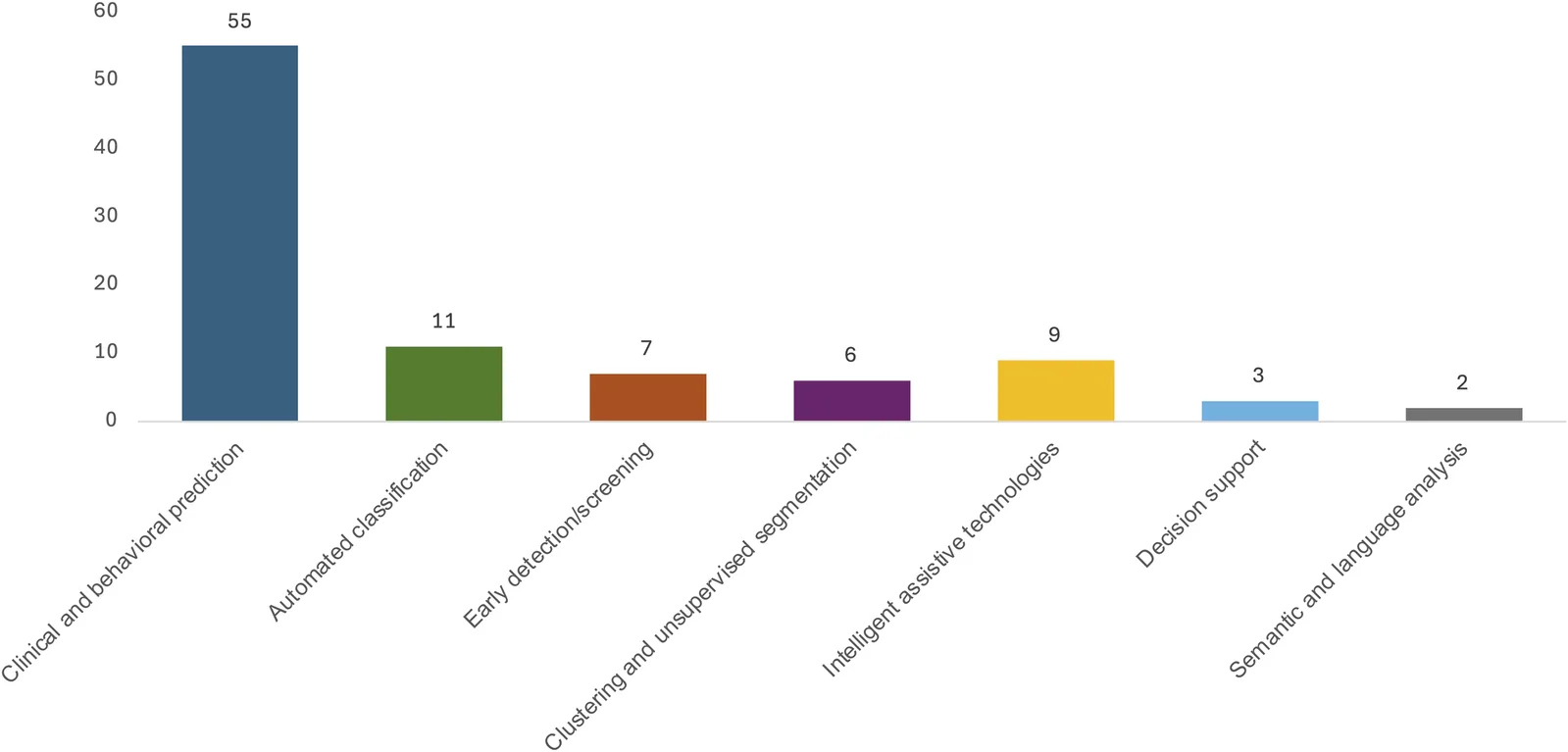
Functional mapping of AI applications addressed in studies.

### Equity, diversity and inclusion considerations

Despite frequent rhetorical references to equity, active inclusion of PWD in AI research remains rare. Only six studies (6.5%) demonstrated participatory approaches such as co-design, usability testing, or user validation. For example, usability testing was conducted during the development of a robotic rehabilitation device in Mexico [105], while a tongue-driven interface explicitly prioritizing accessibility was designed to enhance computer interaction in Fiji [108]. The clarity and inclusiveness of AI-generated health information were evaluated in Canada [50], and functional communication and literacy skills were incorporated to improve diagnostic accuracy for language disorders in American preschool children [39]. Algorithmic bias against neurodivergent individuals was investigated to promote inclusive AI design [29], and user-centered validation was integrated into emotion recognition tools developed for children with Down syndrome in Mexico [104]. Beyond these examples, twelve studies (12.9%) operationalized equity through concrete measures such as adapting tools for vulnerable populations, accounting for sample diversity, or integrating cultural factors [24,41,47,70,85,95,97,109,111,112]. This gap underscores a recurring disconnect between equity as a rhetorical commitment and equity as a research practice, with significant consequences: technologies risk misalignment with user needs, limited adoption, and reinforcement of existing inequalities in use. Furthermore, two studies (2.2%) explicitly addressed equity issues in AI models, albeit from different perspectives. One examined how biological biases embedded in datasets can compromise drug discovery for Alzheimer’s disease and limit global applicability [89], while the other emphasized data representativeness and inclusive modeling in behavioural prediction through facial emotion recognition in children with Down syndrome [104]. Together, these examples illustrate how equity considerations emerge both in biomedical research and in assistive technology development, reinforcing the need for transparent, context-sensitive approaches to mitigate bias and enhance the social relevance of AI for disability.

## Discussion

The study results highlight several major disparities, particularly in terms of the geographic distribution of scientific production, the types of disabilities addressed, the algorithmic approaches prioritized, and the forms of participation of PWD in AI research. By examining these dimensions together, the discussion identifies recurrent patterns observed within the reviewed literature that may constrain the development of artificial intelligence applications that are both inclusive and context-sensitive for PWD. Since this review did not include a formal temporal trend analysis, the findings should not be interpreted as evidence of change or persistence over time.

### Global concentration of AI research for PWD

Results show a pronounced geographic concentration of scientific production in HICs, while LMICs contribute far fewer publications, with China as a notable exception. This imbalance reflects persistent structural disparities in global scientific production, including unequal access to research funding, technological infrastructure, and institutional support for disability-related innovation [112,113]. These patterns also underscore geopolitical inequalities in how disability and assistive technologies are positioned within national and transnational research agendas [114]. Beyond the numerical imbalance, this concentration has systemic implications. AI models developed in HICs are predominantly trained on datasets that reflect the sociocultural, linguistic, and infrastructural realities of resource-rich contexts. When transferred to LMICs, such models risk misalignment with local health systems and lived experiences, potentially reinforcing inequities rather than alleviating them [115]. The limited representation of LMIC contexts raises concerns regarding the transferability and contextual validity of AI-based interventions for PWD. In under-resourced settings, accessibility challenges are further compounded by infrastructural constraints such as limited internet connectivity, scarcity of specialized equipment, linguistic diversity reflected in the absence of localized datasets or multilingual interfaces, and socio-economic barriers related to the high cost of implementation [116]. Evidence from LMICs further shows that even when digital and AI-enabled systems are piloted, sustainability is frequently undermined by unreliable connectivity and electricity, limited equipment, staff turnover, and fragmented governance, reinforcing the gap between resource-rich development contexts and under-resourced deployment environments [114].

Overall, these findings highlight the importance of context-sensitive AI design that integrates local realities, health-system capacities, and sociocultural specificities throughout the development and deployment process. Such approaches that emphasize inclusion, contextual relevance, and lived experience are critical to ensuring that AI systems are designed with and for diverse disability communities [3,113]. Embedding equity as a practical commitment rather than a rhetorical principle will be decisive in transforming AI from a technology that risks reproducing structural inequalities into a meaningful driver of global inclusion and social justice.

### Model trends and methodological gaps

The functional mapping of the 93 included studies reveals a strong predominance of supervised predictive modeling. These approaches are largely applied to biomedical datasets, including neuroimaging, biomarkers, and hospital records with the primary objective of optimizing clinical decision-making and predicting diagnosis. While such models contribute to improved diagnostic precision and clinical efficiency, they simultaneously reinforce a biomedical paradigm that privileges quantifiable impairments and standardized performance metrics over experiential and contextual dimensions of disability, such as QoL, patient-reported outcomes, and the evaluation of real-world system performance following implementation [32,64,69,117]. This narrow, albeit clinically valuable, focus risks overlooking the broader social, cultural, and environmental determinants of disability, particularly for individuals whose lived experiences cannot be adequately captured through biometric or clinical data alone [112,113]. This concern is not hypothetical: decomposition analyses from LMICs, such as Kazemi Karyani et al.‘s study in Iran, demonstrated that disability status independently predicts poor health-related quality of life even after adjusting for income and education, indicating that AI models trained solely on clinical or biometric data will miss disability-driven disparities rooted in social and economic determinants [118]. Morever, the dominance of biomedical datasets often generated in hospital-based, resource-rich settings further limits the contextual relevance of AI systems when applied beyond these environments. Such an orientation stands in tension with the principles of diversity, inclusion, and social responsibility articulated in the Montréal Declaration for Responsible AI, which calls for technologies that account for heterogeneity, accessibility, and social impact rather than performance alone [3].

Another important methodological concern includes small or unrepresentative sample sizes, heterogeneous data sources, and limited external validation. These limitations undermine the generalizability of AI-driven interventions and risk perpetuating inequities when models are deployed across diverse populations and contexts [113,117,119]. Moreover, by privileging hospital-based data and clinical endpoints, this narrow lens channels innovation toward medicalized, institution-centred applications, while marginalizing community-level, social, and environmental interventions. In doing so, it risks reproducing ableist norms and excluding individuals whose disabilities are not readily medicalized or quantified. This clinic-centric pipeline also overlooks programme sustainment constraints that systematically arise in LMIC health systems from usability and hybrid paper-digital workflows to the lack of integration with national programmes which directly limits real-world equity gains [116]. To address these limitations, patient inclusive and participatory frameworks help to consider lived experiences, co-design with PWD, and contextual adaptation as essential components of socially responsive innovation. As technical performance alone does not guarantee equitable outcomes, future AI developments should prioritize context-sensitive, user-informed, and rights-based models that align technological innovation with the lived realities of PWD across diverse sociocultural and geographic settings [112,113].

### Disability typologies: Biomedical bias and underrepresentation of invisible conditions

The typological analysis of studies reveals a marked imbalance in the disability categories targeted by AI applications in healthcare. This distribution reflects a persistent biomedical bias in AI research, privileging disabilities that are readily amenable to technological modeling, such as motor or neurodegenerative conditions, while marginalizing others [112,119]. In contrast, disabilities characterized by subjective, or socially embedded experiences, including many mental health conditions, emotional regulation disorders, and psychosocial disabilities remain largely underrepresented, despite their high prevalence and substantial impact on daily functioning and QoL. This may be due to limited data availability and the inherent challenges in measuring clinically these conditions [29,69]. As a result, non-medicalized or socially situated forms of disability are systematically excluded, constraining the inclusive potential of AI technologies may cause inequities in access to diagnosis, care pathways, and support services [112,119]. In LMIC deployments, these blind spots are further compounded by structural barriers to sustaining digital and AI-enabled interventions including gaps in training, limited availability of devices, and the additional time burden associated with hybrid paper–digital systems which disproportionately affect under-recognized and non-medicalized disability experiences [120]. Scholars therefore emphasize the need for participatory design frameworks and context-sensitive modeling approaches to ensure that AI systems reflect the full spectrum of disability experiences. Moving beyond performance-centric paradigms toward socially responsive and justice-oriented innovation is essential to counteract biomedical dominance and to align AI development with the rights, agency, and lived realities of PWD [112,113,119].

### Equity, diversity and inclusion considerations

This review further highlights a persistent tension between technical performance and social relevance in the development of AI for PWD. In HIC such as the USA, France, and the UK, equity is frequently invoked at a theoretical level but rarely translated into evaluation frameworks, measurable indicators, or sustained participatory mechanisms involving PWD [121,122]. By contrast, several studies conducted in China, Spain, Canada, Iran, and Zambia demonstrated more engaged and pragmatic approaches, placing greater emphasis on accessibility, reduction of clinical burden, and adaptation to local realities. Multiple factors may account for this divergence.

First, research objectives in these contexts often prioritize practical impact, necessitating participatory design strategies and context-sensitive solutions that respond to infrastructural, cultural, and socioeconomic conditions [123]. Second, data-centric AI research typically overlooks approaches that enable deeper engagement with lived experience, intersecting vulnerabilities, and syndemic factors shaping disability and health outcomes, such as qualitative and mixed-methods approaches [112]. Together, these findings suggest that methodological choices and contextual imperatives play a decisive role in moving equity from rhetorical commitment to research practice. These examples further underscore the potential of context-sensitive AI to address the specific needs of marginalized populations when equity is treated as an actionable design principle rather than an abstract ethical goal [121,124]. Systematic evidence from LMICs indicates that equity commitments translate into impact only when sustainability enabling factors are in place, such as stakeholder engagement, governance alignment, recurrent financing, and continuous capacity-building across the intervention life-cycle [120]. Beyond issues of accessibility and representation, AI systems may also generate unintended harms for people with disabilities. These include algorithmic bias, discriminatory decision-making, exclusion resulting from inaccessible interfaces, and risks associated with the use of non-representative datasets. Such concerns have been documented across multiple AI domains and warrant greater attention in future disability-inclusive AI research [125].

Conversely, open and customizable AI systems may help reduce structural disparities by enabling local adaptation, provided that robust participatory frameworks, accountability mechanisms, and safeguards are in place to prevent new forms of exclusion or harm [116]. Pragmatic participatory approaches including co-design with PWD, community-based validation, and the integration of local expertise are therefore essential to ensuring that AI technologies are not only technically effective but also socially relevant and culturally appropriate. Addressing these inequities may benefit from stronger international collaboration and targeted investments supporting inclusive research. However, the present review does not provide evidence for a causal relationship between funding mechanisms and inclusivity outcomes. International partnerships should prioritize capacity-building in LMICs, notably through training programs for local researchers, investment in accessible research infrastructure, and fair access to datasets. Without such structural commitments, AI-driven health innovation risks reproducing existing power asymmetries rather than contributing to inclusive and just technological futures for PWD.

## Limitations

This scoping review presents some limitations. First, one key limitation is that our review did not explicitly target community-based AI initiatives in health, as most, if not all, of the studies we examined were conducted in clinical settings. Second, the exclusion of non-English publications and grey literature such as institutional reports, theses, and both indexed and non-indexed conference proceedings, may reinforce publication bias and underrepresent practice-oriented or context-sensitive innovations, especially those emerging from the LMICs. Additionally, the omission of preprints and conference papers may also result in overlooking emerging prototypes and experimental tools that are not yet formally published but hold significant relevance for the field. The proportion of studies reporting participatory approaches may also be underestimated because conference proceedings, non-indexed publications, grey literature, and studies published in languages other than English or French were excluded from this review.

Finally, the use of broad disability categories to synthesize a heterogeneous body of literature is another limitation. Although a condition-specific breakdown was provided, grouping neurodegenerative disorders, neurodevelopmental conditions, intellectual disabilities, and specific learning disorders within a broad cognitive category may obscure important differences in etiology, clinical trajectory, AI applications, accommodation needs, and ethical considerations, including consent capacity. Consequently, findings for this category should not be interpreted as applying uniformly to all included conditions. More importantly, they highlight key directions for future research: expanding linguistic and geographic coverage, systematically integrating grey literature, improving transparency and reporting standards, and ensuring the active participation of PWD as co-creators rather than passive subjects.

### Implications of AI in disability research

The findings of this review reveal three major domains where AI carries significant implications for disability research: scientific, clinical, and policy-related.

#### Scientific implications.

AI has demonstrated strong potential in predictive modeling (62.4% of studies), particularly in analysing complex biomedical datasets [29,40,92]. However, this dominance narrows research priorities and limits interdisciplinary innovation. Key recommendations include: 1) diversifying applications beyond diagnosis to include interaction, communication, and inclusive support systems [33,35,87]; 2) ensuring algorithmic transparency [60,104]; 3) promoting interdisciplinary collaboration between computer science, rehabilitation, psychology, and social sciences to counter dominant biomedical approaches [61,62]. To transform AI into a tool for inclusive innovation, it is essential to: strengthen participatory methodologies, diversify research contexts, and implement explicit equity evaluation frameworks [123]. Only under these conditions can AI [126] evolve from a technical solution into a socially responsible instrument, aligned with the 2030 Sustainable Development Goals and beyond, and the lived realities of PWD [101]. One actionable pathway is embedding AI within primary health care (PHC) infrastructures: as Koolivand and Amini demonstrated during COVID-19 in Iran, PHC-based measures, including community health worker networks reached people with disabilities whom hospital-centric systems missed. AI for disability inclusion will fail if deployed only in specialized tertiary centers; PHC-integrated, decentralized AI architectures are a necessary but underexplored direction [126]. Funding agencies and global institutions must prioritize inclusive policies that direct resources toward equity-deserving groups, including those marginalized by gender, disability, and other intersectional identities, as well as underrepresented regions. This shift is essential to building a more equitable and diverse evidence base.

#### Clinical implications.

AI is primarily deployed in specialized hospital settings in high-income countries [28,50,99], limiting its transferability to low-resource environments. To enhance clinical relevance, an emphasis on tool adaptation, especially in LMICs, considering infrastructure and accessibility constraints, is essential. Furthermore, validating AIf models with diverse populations, including people with different disabilities, is critical to enabling personalized care and inclusive rehabilitation. This process must also confront methodological challenges, such as small sample sizes and the absence of external validation, before these tools can be responsibly deployed in clinical settings.

#### Policy and governance implications.

The geographic imbalance in scientific production reflects deeper structural inequalities in funding and research priorities [28]. Our study findings demonstrate the need to promote inclusive governance, by strengthening LMIC representation through targeted funding and international collaboration [105], in addition to operationalizing more effectively equity through concrete measures, such as sample diversity, cultural adaptation, and digital inclusion [31,32,36]. These ethically based practices further align with the United Nations Convention on the Rights of Persons with Disabilities, World Health Organization, and Organization for Economic Co-operation and Development (OECD) guidelines to ensure responsible and disability-inclusive AI use [51,68].

## Conclusion

This scoping review shows that AI is rapidly reshaping disability health-related research, with predictive and diagnostic applications dominating the field. These approaches highlight AI’s technical capacity to process complex biomedical datasets and optimize care, yet they also expose persistent shortcomings: methodological fragility, limited integration of equity principles, and the marginal involvement of PWD. Such gaps raise concerns that AI may reinforce existing inequalities rather than reduce them. At the same time, the review identifies promising innovations in assistive technologies, inclusive communication systems, and context-sensitive applications developed in the LMICs. These contributions underline that AI cannot be treated as a purely technical solution but must be grounded in cultural, social, and ethical realities. The way forward requires a paradigm shift: moving from fragmented, technically driven projects to inclusive, participatory, and context-aware innovation. By ensuring transparency, fostering interdisciplinary collaboration, addressing North-South asymmetries, and involving PWD as co-creators, AI can evolve into a transformative tool for autonomy, equity, and social justice. The future of AI in disability-related health research depends not only on improving algorithms but also on rethinking how innovation is designed, governed, and shared, ensuring it upholds the rights, dignity, and well-being of PWD globally. The principle of *“Nothing about us without us”* must guide the design, development, and implementation of technologies intended for PWD. By centering their voices and leadership, we can transform AI from a mere scientific and clinical tool into a catalyst for social justice [120]. This approach ensures that innovation truly reflects and responds to the needs and realities of the disability communities. By synthesizing these dimensions, the review will: 1) guide funding agencies, policymakers and researchers in prioritizing underrepresented disability groups, low-resource regions, and inclusive research practices aligned with the *Montréal Declaration* [4] and the Sustainable Development Goals (SDG 3, 9, 10) [6]; 2) provide a reference framework for developers seeking to embed transparent reporting (e.g., model cards, open data) and user-centered design (“nothing about us without us”) into future AI products; and 3) identify methodological shortfalls such as small sample sizes, opaque model descriptions, and lack of external validation that must be addressed before clinical or societal deployment. In sum, this review does not merely catalogue existing work; it offers an evidence-based roadmap for advancing AI toward inclusive, accountable, and equitable technologies that uphold the rights and well-being of all persons with disabilities.

## Supporting information

S1 Text Search strategy. (DOCX)

S2 Text Table of characteristics of included studies. (DOCX)

S1 Checklist PRISMA checklist. (DOCX)

S1 Data Data. (XLSX)

## Abbreviations

AI: Artificial Intelligence
BCI: Brain-Computer Interface
fNIRS: Functional Near-Infrared Spectroscopy
MCI: Mild Cognitive Impairment
SVMs: Support Vector Machines
CNN: Convolutional Neural Network
CNN-LSTM: Convolutional Neural Network - Long Short-Term Memory
QoL: Quality of Life
AD: Alzheimer’s Disease
ASD: Autism Spectrum Disorder
EEG: Electroencephalography
ERPNet: Event-Related Potential Network
IMU: Inertial Measurement Unit
ML: Machine Learning
DL: Deep Learning
NLP: Natural Language Processing
RL: Reinforcement Learning
PROMs: Patient-Reported Outcome Measures
