# Supplementary material for: Contribution of artificial intelligence research to the development of inclusive applications for people with disabilities: A scoping review (2015–2025)
Source: PLOS Digit Health. 2026 Oct 15;5(10):e0001749. doi: 10.1371/journal.pdig.0001749 (PMC13671530; doi:10.1371/journal.pdig.0001749)
Supplement: Supplementary file 1 [file pdig.0001749.s001.docx]

**Search terms**

**Search Keywords**

Search component

Impair* or Functional disability or Genomic disorder or Motor disability or Neurological disability or Visually impared* or Mobility impared* or Amputee* or disabled persons or disabled, physically or handicapped* or handicapped, physically or deficiency, mental or development disorder, intellectual or development disorders, intellectual or mental deficiency or mental retardation or developmental disabilities or developmental disability or ambulation difficulties or ambulation difficulty or ambulatory difficulties or ambulatory difficulty or difficulties, ambulation or difficulties, ambulatory or difficulty, ambulation or difficulty walking or limitation, mobility or mobility limitation or mobility limitations or walking, difficulty or visual impairment or disability, communication or communicative dysfunction or Rehabilitation

People with Disabilities

**AND**

Artificial Intelligence or Robot* or Robotics or Algorithm* or machine learning or deep learning or Incremental learning or reinforcement learning or augmented reality or Telerehabilitation or transfer learning or virtual environment* or virtual reality or computer vision or neural network* or sentiment analysis or natural language processing or transformer* or Internet of Things or IoT or neurorobotic* or Fuzzy or intelligence, computational or intelligence, machine or knowledge acquisition* or knowledge representation* or machine intelligence or reasoning, computer or representation, knowledge* or system, computer vision or systems, computer vision or vision system, computer or vision systems

Artificial Intelligence

**AND**

Computer program development* or Program design* or Applied research* or Systems research* or Patient-Based Outcome Research* or health care research* or health services evaluation* or action research* or medical care research* or research, health care* or research, consumer-driven community-based*

Research Developement
