# Supplementary material for: Contribution of artificial intelligence research to the development of inclusive applications for people with disabilities: A scoping review (2015–2025)
Source: PLOS Digit Health. 2026 Oct 15;5(10):e0001749. doi: 10.1371/journal.pdig.0001749 (PMC13671530; doi:10.1371/journal.pdig.0001749)
Supplement: Supplementary file 2 [file pdig.0001749.s002.docx]

**Characteristics of included studies**

| **Authors (year)**  **Country** | **Study objective** | **AI Application** | **Disability type** | **Barriers for PWD’ inclusion** | **Facilitators for the inclusion of PWD** | **Equity considerations** | **Implications (policy, practice, research)** |
| --- | --- | --- | --- | --- | --- | --- | --- |
| N°1  Abbott (2017)  USA [1] | To develop a supplemental process for identifying some overlooked but gravely ill applicants. | Prediction | Mobility | Small sample size | Not addressed | Sample diversity | Identification of gravely ill Social Security disability applicants by analysing the unstructured electronic health records. |
| N°2  Akin-Bulbul (2022), Turkey [2] | To develop and test a Machine Learning (ML) based Decision Support System (DSS) to predict young children with autism spectrum disorder (ASD) using eye-tracking data. | Prediction | Communication | Limited sample size and gender proportion differences between groups. False-negative cases. | Not addressed | Not addressed | Not addressed |
| N° 3  Akter (2022)  USA [3] | To handle data imbalance in predicting fatality and risk factors in Alzheimer's patients with COVID-19. | Prediction | Cognition | Addressed the challenges of imbalanced data, particularly in the context of predicting fatality rates and risk factors for AD patients with COVID-19. | Not addressed | Not addressed | To highlight the importance of utilizing advanced deep learning models in clinical practice to enhance accuracy and performance in predicting patient outcomes. |
| N°4  Almufareh  (2023), Saudi Arabia [4] | To develop an attention-based mechanism that employs the vision transformer approach to detect Alzheimer’s using MRI images. | Prediction | Cognition | Not addressed | Not addressed | Not addressed | Superior performance, outperforming other published methods when applied to the Kaggle dataset. |
| N° 5  Alves (2022), USA [5] | To validate a machine learning model to estimate Expanded Disability Status Scale (EDSS) scores for multiple sclerosis patients using clinical notes from neurologist visits. | Prediction | Mobility | Using information extracted solely from clinical notes, variations in documentation practices across different geographic locations or care settings, and the need for generalizability to multiple data sources. | The use of machine learning to estimate Expanded Disability Status Scale (EDSS) scores from clinical notes facilitated the generation of eEDSS scores for a large number of encounters. | Not addressed | To highlight the importance of using machine learning to estimate disability scores in real-world data sources, |
| N°6  Ames (2019), USA [6] | To apply artificial intelligence-based hierarchical clustering to optimizes performance for adult spinal deformity surgery. | Clustering | Mobility | Small sample size | Not addressed | Sample diversity | To facilitate treatment optimization by educating surgeons on which treatment patterns yield optimal improvement with lowest risk. |
| N°7  Assaf (2023), Fiji [7] | The main objective of the article is to present the development of an optimized tongue-driven system using artificial intelligence. | Physical aide | Mobility | Challenges related to the accuracy, responsiveness, and integration of the system with other peripherals. | Facilitate persons with disabilities in interacting with computers by providing an assistive device that translates tongue movements into user-defined commands. | Promoting equity in access to technology and computer interaction | To prioritize the development of cost-effective, responsive, and accurate assistive technology to enhance the quality of life for individuals with disabilities, particularly in the context of computer interaction. |
| N°8  Baldo (2023), Italy [8] | To provide an analysis of clinical records obtained from subjects with DS and study their association with Intellectual disability. | Prediction | Cognition | Reliability of feature importance score | Not addressed | Sample diversity | To highlight the accuracy to identify variables likely involved in cognitive impairment in Down syndrome |
| N°9  Bellemo (2019), Zambia [9] | To detect vision-threatening diabetic retinopathy and diabetic macular oedema among an African population with diabetes using AI. | Classification | Vision | Data was self-reported by patients, low sample size, usability of the AI, deployment of the system. | Not addressed | Sample diversity | To highlight AI system to be clinically acceptable to detect referable diabetic retinopathy in this population. |
| N°10  Ben-Shalom (2016), USA  [10] | To assess the feasibility of using existing claims-based algorithms to identify Medicare beneficiaries living in the community with a disability. | Prediction | General disability / Unspecified | Non-inclusion of all populations with disabilities. | Not addressed | No | Application to Social Security Disability Insurance (SSDI) Claimant Medical Claims Data. |
| N°11  Benabid (2019), France [11] | To demonstrate a proof of concept for a brain-machine interface system that allows a tetraplegic patient to control a four-limb exoskeleton using signals from epidural electrocorticography. | Physical aide | Mobility | Exclusion of one of the two participants recruited due to technical problems with the implants. | Not addressed | No | To highlight the feasibility of controlling a four-limb exoskeleton using brain signals decoded from epidural ECoG in a patient with tetraplegia. |
| N°12  Berg (2024), Norway [12] | To develop and validate prediction models for disability and pain 12 months after lumbar disc herniation surgery. | Prediction | Mobility | Data validation | Not addressed | Nation-wide registry included | To show the power of machine learning in the related studies. |
| N°13  Bertoncelli (2020), France [13] | To predict health conditions in children with cerebral palsy. | Prediction | Cognition | Small sample size , external validation of the model. | Not addressed | Sample diversity | To help doctors’ decision-making process regarding patient prognosis. |
| N°14  Bertoncelli (2020), France  [14] | To develop and validate a machine learning model for gastrostomy placement in adolescents with developmental disabilities and cerebral palsy. | Prediction | Mobility | Lack of standardized criteria, follow-up studies needed. | Not addressed | Sample diversity | To highlight efficiency of the model for the prediction. |
| N°15  Bhagat (2020), USA [15] | To predict the extent of motor recovery. | Prediction | Mobility | Generalizability | Not addressed | Not mentioned any separation between patients. | Evidence of neuroplasticity and cortical reorganization following BMI-mediated rehabilitation therapy. |
| N°16  Bhagwat (2019), USA and Canada [16] | To predict the severity in Alzheimer's disease. | Prediction | Cognition | Clinical Heterogeneity; longitudinal Data Limitations; model Complexity; and computational Resources. | Not addressed | No | Multimodal data Integration and continuous monitoring. |
| N°17  Bochniewicz (2017), USA  [17] | To present a novel method for separating upper extremity functional use from non-functional movement after stroke using a single wrist-worn accelerometer. | Classification | Mobility | Data was self-reported by patients, low sample size, low measured features. | Not addressed | Sample diversity | To distinguish with good accuracy the proportion of movements that fall into the functional use category. |
| N°18  Bragg (2023), USA [18] | To predict the Subjective Visual Vertical (SVV) and Ocular Vestibular Evoked Myogenic Potential (o-VEMP). | Prediction | Hearing | Small sample size, generalizability, the complexity of the data and the need to carefully select and preprocess. | Not addressed | No | To enhance diagnostic accuracy and potentially improve patient outcomes. |
| N°19  Brandsen (2024), USA [19] | To investigate the prevalence and degree of AI-bias against neurodivergent individuals. | Bias analysis | Cognition | Technical limitations, limited analysis to English texts. | Not addressed | Sample diversity | To highlight the importance of “de-biasing” AI so that autistic and other neurodivergent people do not experience discrimination in job applications or other AI-based processes. |
| N°20  Briend (2023), France [20] | To evaluate the classification performance of acoustic features of the voice in children with autism spectrum disorder (ASD) with respect to a heterogeneous control group. | Prediction | Cognition | Small sample size and the need for replication with larger sample sizes. | Use of voice acoustics as an objective diagnostic tool for ASD. | No | To development an early diagnostic biomarker specific to Autism. |
| N°21  Bryan (2023), USA [21] | To investigate the use of brain co-processors to achieve goal-directed closed-loop neurostimulation for optimizing rehabilitation goals after injury. | Prediction | Multiple (neurological impairment) | Complexity of predicting neural responses to stimulation over long time scales, matching the dimensionality of the brain with current sensor and stimulator technologies. | Not addressed | No | To develop neural co-processors for learning complex adaptive stimulation policies for a variety of neural rehabilitation and neuroprosthetic applications. |
| N°22  Cantillo-Negrete (2021), Mexico [22] | To Evaluate of the feasibility of an intervention with a BCI based on motor intention coupled with a robotic hand orthosis for rehabilitation after an upper limb stroke. | Physical aide | Mobility | Limited duration of intervention sessions, small sample size | Not addressed | Involving usability testing with PWD to ensure that AI solutions meet their needs, as well as their preferences. | Not addressed |
| N°23  Cartwright (2023), USA [23] | To describe how machine learning techniques can assist with MS diagnosis, personalize treatment plans, and optimize rehabilitation strategies for improved patient outcomes in order to be able to apply this technology to patient care. | Prediction | Mobility | Technology limitations | Not addressed | No | ML’s effectiveness in locating lesions, diagnosing MS, gauging level of disability, and assisting with rehabilitation. |
| N° 24  Chen (2023), England [24] | To identify the most important predictors for these outcome measures in stroke rehabilitation. | Prediction | Cognition | Small sample size | Not addressed | No | To highlight feasibility of machine learning to predict postintervention PROMs for activities and participation in chronic stroke. |
| N° 25  Cheng (2020), Singapore [25] | To evaluate of the feasibility and clinical application of the system which incorporates activities of daily living (ADL)-oriented tasks for stroke rehabilitation. | Prediction | Mobility | Small sample size, equity considerations | Gamification |  | To improve motor function in stroke survivors. |
| N°26  Cho K. (2023), South Korea  [26] | To identify determinants of depression in caregivers of dementia patients using machine learning algorithms and statistical models. | Prediction | Cognition | Not addressed | Not addressed | Help in developing policies to improve the mental health of this vulnerable population. | To improve the mental health of dementia caregivers by policy development. |
| N°27  Connell (2021), New Zealand  [27] | To explore factors influencing the implementation of PREP2 as perceived by staff. | Prediction | Mobility | Initial resistance to change and the need for extensive training. | Having a champion advocate. | No | To explore modifiability of factors. |
| N°28  Crowson (2020), Canada  [28] | To develop ‘Auto Audio’ – a novel deep learning proof of concept model that accurately and quickly interprets diagnostic audiograms. | Prediction | Hearing | Standard format for recording and storing results | Not addressed | Sample diversity | To enable automatic and accurate audiogram interpretation using deep learning. |
| N° 29  de Vries (2023), Netherlands  [29] | To develop an automatic stress detection system suitable for real-time use and to assess if the system generalizes to people with ID. | Prediction | Cognition | Small sample size, self-reported data | Not addressed | Sample diversity | The personalized model can detect stress in people with and without disabilities |
| N°30  Dosso (2024), Canada  [30] | To compare the quality of information about Alzheimer's Disease (AD) and other dementias provided by ChatGPT with that provided by official AD organizations. | Prediction | Cognition | The varying quality and format of information | ChatGPT to improve health literacy and provide support for the patient-physician relationship without endorsing specific products. | Tools are accessible and understandable to a broad audience, including individuals with low health literacy. | Not addressed |
| N°31  Farhadian (2015), Iran [31] | To analyse the potential of AI for estimation of hearing impairment among industrial workers. | Prediction | Hearing | Self-reported data, small sample size | Not addressed | No- Only men were considered | To provide reliable and comprehensible information for occupational health and medicine experts. |
| N°32  Frazier (2023), USA [32] | To develop social and cognitive stimulus paradigms that could be paired with webcam collection and artificial intelligence algorithms to measure key neurocognitive process. | Prediction | Cognition | Small sample size | Not addressed | Sample diversity | To capture individual and between group differences in neurobehavioural function. |
| N°33  Fromm (2022), USA [33] | To enhance the classification of aphasia through statistical analysis using connected speech. | Prediction | Communication | The time-consuming nature of transcription, the large number of variables and measures. | Not addressed | No | To highlight the importance of a more systematic approach to discourse measurement and analysis in aphasia studies. |
| N°34  Gao (2023), China [34] | To Investigate core Alzheimer's disease (AD) biomarkers in a Han Chinese cohort. | Prediction | Cognition | Single-center data source, medium sample size, lack of measurements, absence of individuals with non-Alzheimer's dementia for comparison. | Not addressed | Focus on the need for accurate AD biomarkers in the Chinese Han population, importance of regional guidelines for AD diagnosis, potential for global multicenter trials in China. | To reveal accurate Alzheimer disease biomarkers, exploration of new biomarkers related to neuroimmune dysregulation, synaptic dysfunction, and blood-brain barrier damage. |
| N°35  Geng (2024), China [35] | To review how machine learning methodologies are being deployed in drug discovery for Alzheimer’s Disease (AD), specifically focusing on target identification and intervention strategies. | Prediction | Cognition | High cost of traditional drug discovery, the complexity of factors, the presence of the blood-brain barrier (BBB) and heterogeneity od data. | Not addressed | No | To accelerate the drug discovery process, improving and research priorities include addressing the current limitations of ML techniques and further integrating AI/ML approaches to improve drug discovery outcome. |
| N°36  Gharbi-Meliani (2023), Europe  [36] | To develop unsupervised machine learning and longitudinal data to identify probable dementia cases. | Prediction | Cognition | Lack of clinical diagnosis in the population ageing and the potential for underestimation of dementia cases if relying solely on self-reported data. | Not addressed | No | To aid the early detection and better understanding of dementia progression. |
| N°37  Grassi (2019), USA [37] | To develop a new machine learning algorithm to allow a 3-year prediction for conversion to AD in subjects diagnosed with mild cognitive impairments. | Prediction | Cognition | Small sample size | Not addressed | Sample diversity | To improve recruitment in clinical trials and to prescribe new and newly emerging early interventions more selectively to high AD risk patients. |
| N° 38  Guan (2019), China [38] | To identify the key factors and research trends of AD for guiding furthermore effective studies. | Clustering | Cognition | Potential bias in the data | Not addressed | Sample diversity | To reveal the hotspots and trends of Alzheimer's disease. |
| N°39  Hart Kamber (2021), USA [39] | To precise recognition of disability arising from neuropsychiatric diseases. | Prediction | Cognition | Self-reported data, small sample size | Not addressed | Sample diversity | To characterize agitation-related symptoms through natural language processing may provide new tools for understanding agitated behaviours and their relationship to delirium. |
| N°40  He (2021), USA  [40] | To develop deep multimodal learning models for the early prediction of cognitive, language, and motor deficits using multimodal MRI and clinical data. | Prediction | Cognition | Methodological limitations, external validation | All subjects were scanned during natural sleep without the use of any sedation after being fed and swaddled | Participants inclusion criteria considered all features | To demonstrate the value of multimodal MRI features as potential biomarkers for prediction of later neurodevelopmental deficits. |
| N°41  Hill (2022), USA  [41] | To predict customer characteristics and vocational rehabilitation services outcomes. | Prediction | Mobility | Small sample size | Not addressed | Sample diversity | To consider implementing early, targeted interventions for newly identified at-risk groups of vocational rehabilitation customers. |
| N°42  Hruby (2017), Australia [42] | To design of a treatment algorithm for bionic reconstruction of the hand. | Physical aide | Mobility | Logistical problems | Not addressed | No | To extraction information on existing weak muscle signals and improvement of prosthetic function. |
| N°43  Hu (2021), China [43] | To develop a learning model based on a Bayesian network for inclusive dance training to analyse the physical development of students with developmental disorders of the musculoskeletal system. | Classification | Mobility | Small sample size | Not addressed | No- The dataset is not comprehensive | To improve students’ physical fitness and, consequently, their overall coordination of movements. |
| N° 44  Imbiriba (2023), USA [44] | To predict imminent aggressive behaviour in inpatient youths with autism before it occurs. | Prediction | Cognition | The reluctance of some participants to wear the biosensor. | Not addressed | No | To highlight the potential for wearable biosensors and machine learning to predict and possibly prevent aggressive behaviours in youths with autism. |
| N°45  Ingelse (2022), Portugal [45] | To monitor gait patterns in people with neurological conditions by assessing the benefits of personalisation on ML models. | Prediction | Mobility | Data limitation, annotation process is prone to errors. | Not addressed | Limited an imbalanced data | To improve personalized model and overall accuracy. |
| N°46  Janssen (2021), Netherlands [46] | To investigate the associations between preoperative physical performance measures and postoperative outcomes. | Prediction | Mobility | Data Limitations (presence of missing data), Single-Center Data, Complexity of data. | Not addressed | No | Not addressed |
| N°47  Justice (2019), USA [47] | To identify child- and family-level characteristics most strongly associated with the clinical identification of language disorders in preschool-aged children. | Prediction | Communication | Limited impact of home environment measures and poor prediction accuracy with basic background information. | Inclusion of functional communication and literacy skills. | No | To enhance the identification of children with language disorders by incorporating multiple sources of information and improving classification accuracy. |
| N° 48  Kang (2019), USA [48] | To detect the potential risk factors of Alzheimer's disease (AD) from two perspectives. | Risk factors detection | Cognition | Data limitation | Not addressed | No | To identify important risk factors on cognitive decline and the transition from cognitive normal to Alzheimer’s disease. |
| N° 49  Kang (2021), China [49] | To propose a novel EL CNN system that integrates 11 of the best validation accuracy 2D slice-level models from three CNNs for final classification. | Prediction | Cognition | Limited neuroimaging data availability, Data leakage issues in longitudinal studies. | Not addressed | No | To drive advancements in Alzheimer's disease diagnosis, improve patient outcomes. |
| N°50  Kanzler (2022), Belgium [50] | To explore the feasibility of predicting upper limb rehabilitation outcomes in individuals. | Physical aide | Mobility | Small sample size | Not addressed | No | To establish the feasibility of an individualized prediction of upper limb neurorehabilitation outcomes. |
| N°51  Kim (2022), South Korea [51] | To examine the psychometric properties and establish the diagnostic validity of the K-ADOS-2 across all modules with a larger number of participants. | Prediction | Hearing | Unbalanced gender distribution among participants, and limited information about the OD group. | Not addressed | No | Not addressed |
| N° 52  Klein (2018), USA [52] | To characterize the hearing environment of hearing-impaired older adults, with and without hearing aids. | Prediction | Hearing | Study including only the elderly; the time between hearing aid fitting and assisted environment assessment too short. | Auditory environment; speech environments and social participation | No | To highlight the feasibility of the system to objectively characterize real-world hearing environments and the effect of hearing aids older adults with hearing disabilities. |
| N° 53  Lee (2023), South Korea [53] | To identify effects of altered functional connectivity on motor imagery brain–computer interfaces based on the laterality of paralysis in hemiplegia patients. | Classification | Mobility | Computational limitations, sample size limitations. | Not addressed | No | To improve motor control in patients with physical disorders, as the connectivity between both brain hemispheres. |
| N°54  Lindsay (2021), USA [54] | To understand Alzheimer's disease-related language disorders and their cognitive sub-processes through statistics on multilingual NLP characteristics. | Classification | Communication | Small sample size | Not addressed | No | To demonstrate the application of generalizable language features. |
| N°55  Lombardi (2016), Italy  [55] | To describe the sociodemographic, clinical, and functional characteristics of a representative sample of service users in Italy. | Prediction | Cognition | The single support provider | Not addressed | Sample diversity | To provide support activities aligned to the specific personal needs and goals. |
| N° 56  Mahmut (2022), Romania [56] | To develop an automated screening method for Speech Sound Disorder (SSD) in preschool children’s topics. | Prediction | Communication | Not addressed | Using mobile technology to maintain anonymity and combat the adversity of uninformed parents facing speech therapy/pathology. | Develop an application to assist speech therapists in their laborious and time-consuming activity. | Not addressed |
| N° 57  Marrie (2021), Canada [57] | To develop and internally validate an algorithm for disability status in Multiple Sclerosis using administrative. | Prediction | Mobility | Data limitation, the annotation process is prone to errors. | Not addressed | No | To prove effectiveness of using an algorithm for disability in Multiple Sclerosis (MS) using administrative data that may support population-based studies. |
| N°58  McCombe (2022), England  [58] | To optimizen the Algorithm Development. | Prediction | Cognition | Real-World Constraints; User-Friendly GUI Tool. | Not addressed | No | To enable stakeholders to set budget limits and assessment time costs based on their expertise and financial budgets. |
| N°59  McCombe (2021), USA[59]] | To develop a cost-sensitive algorithm and optimize assessment time. | Prediction | Cognition | Complexity in clinical settings, time ionstraints, Data Integration challenges. | User-Friendly Tools | Accessibility and standardization | To encourage the development of additional methods to optimize healthcare delivery. |
| N°60  Meskers (2022), Netherlands [60] | To address the feasibility, reliability, and internal validity of natural language processing (NLP) for automated functional assessment of hospitalized COVID-19 patients. | Physical aide | Mobility | Class imbalance | The study demonstrated the potential of computational language modeling to classify categories and their levels | No | To aid in personalized rehabilitation strategies and improve patient outcomes |
| N°61  Moreno (2023), Mexico [61] | To detect and analyse facial emotions in children with Down Syndrome to predict their emotions throughout a dolphin-assisted therapy. | Decision- making | Cognition | Data limitation | Not addressed | Sample diversity | To Integrating EEG signals into a CNN architecture can enhance learning and decision-making capabilities. |
| N°62  Nikam (2021), India [62] | To identify potential biomarkers of inherited metabolic disorders in children with intellectual disability using tandem mass spectrometry. | Prediction | Cognition | Small sample size and non-inclusion of other neuro-developmental conditions. | Not addressed | Sample diversity | To identify specific metabolite markers in intellectual disabilities and correlate them with neurological features. |
| N° 63  Noel (2022), England [63] | To investigate the updating of peri-personal space (PPS) in individuals with autism spectrum disorder (ASD) during social contexts. | Prediction | Communication | The study highlighted the challenges in integrating information across sensory modalities and the difficulties in modulating multisensory integration in individuals with ASD during social contexts. | Not addressed | No | To understand the neural mechanisms underlying sensory processing differences in autism spectrum disorder (ASD) and the impact of social contexts on multisensory integration. The research may inform interventions and support strategies for individuals with ASD. |
| N°64  Ogawa (2023), Japan [64] | To provide hypotheses on the causes of brain functions in developmental dyslexia (DD) by constructing and analysing a simple computational model of visual information processing using a deep generative model. | Prediction | Cognition | Computational limitations | Not addressed | Sample diversity | To prove the power of the method. |
| N°65  Olivares-Gil (2022), Italy [65] | To examine Automatic Speech Recognition (ASR) technology for Parkinson’s disease. | Prediction | Communication | Small sample size | Not addressed | No | To investigate for treating dysarthria in a larger sample |
| N°66  Oscar (2017), USA [66] | To develop of a semi-automated text coding method and use a content analysis of Alzheimer’s disease. | Classification | Cognition | Data limitation | Not addressed | Sample diversity | To highlight the effectiveness of technique to questions in social gerontology related to social media |
| N°67  Panganiban (2022), USA [67] | To examine the predictors of language gains for children with autism spectrum disorder (ASD). | Prediction | Cognition | Small sample size | Not addressed | No | To identify children who are likely to benefit most from specific interventions and adjust their therapeutic approaches accordingly. |
| N° 68  Schilling (2023), Germany  [68] | To investigating the application of predictive coding and stochastic resonance in auditory phantom perception. | Prediction | Hearing | Lack of a clearly defined theoretical framework and the complexity of developing appropriate analysis methods that can be generalized to understand neural processes. | Not addressed | No | To help understand and simulate cognitive and neural processes |
| N°69  Shehzad (2022), Canada [69] | To develop a supervised machine learning algorithm to classify dementia stages. | Prediction | Mental | Data Imbalance, Variability in Symptoms. | Not addressed | No | To highlight real-world data utilization |
| N° 70  Steinheimer (2020), Switzerland [70] | To establish video-based stepwise comparison as a fine-grained, reliable and efficient rating method of motor dysfunction using algorithmic support. | Clustering | Mobility | A full validation of the concept has not been investigated | Not addressed | Sample diversity | To highlight that set wise comparison is an efficient, valid and reliable method that can be applied to larger datasets. |
| N°71  Tham (2021), sisngapore [71] | To develop a single-modality, retinal photograph-based deep learning algorithm for detecting disease-related visual impairment and to validate its performance. | Prediction | Vision | Small sample size | Not addressed | No | To expand the capabilities of existing diabetic retinopathy screening programs by identifying other eye diseases. |
| N°72  Tuechler (2020), Australia [72] | To develop and validate an automatic linkage approach that translates information derived from patient-reported outcome measures. | Prediction | Mobility | Algorithmic model not precise enough, small sample size | Not addressed | No | To develop approaches for ICD-11 functional property classification based on ICD-11 in everyday clinical practice. |
| N°73  Tunthanathip (2021), Thailand  [73] | To assess the predictability of ML for the functional outcomes of pediatric Traumatic brain injury. | Prediction | Mobility | Possible bias from confounding factors | Not addressed | Sample diversity | To predict functional outcomes and counselling prognosis in generalpractice of pediatric Traumatic Brain Injuries (TBIs). |
| N°74  Valcarcel (2018), USA [74] | To develop an automatic segmentation approach, adapted from a T2L segmentation algorithm. | Prediction | Cognition | Data limitation | Not addressed | No | To highlight the model effectiveness for segmenting T1 black holes in patients with Multiple sclerosis. |
| N°75  Vaucheret (2020), Argentina [75] | To explore the third-party punishment behaviour in a population of pediatric participants with developmental disorders older than 9 years of age, in an ecological setting. | Sentiment analysis | Cognition | AI does not identify specific positive and negative emotions | Not addressed | considered nationality | To provide insight that can help provide effective interventions to improve outcome. |
| N° 76  Verma (2022), England [76] | To investigate how ML can predict outcomes among patients with non-specific neck and/or low back pain. | Prediction | Mobility | Not mentioned | Not addressed | No | To increase the precision of clinical decision-making and thereby improve patient outcomes in health conditions that are difficult to assess with objective measurements, such as non-specific musculoskeletal pain and mental disorders. |
| N°77  Wategaonkar (2022), India  [77] | To explore different methodologies and techniques for sign language recognition, focusing on word-level and sentence-level recognition. | Prediction | Hearing | Complexity and computational resources, the need for precise domain knowledge labeling, and challenges related to collecting and processing large-scale datasets. | Using multi-modal approaches to improve sign language recognition accuracy. | Bridge the communication gap between people with hearing and speech disabilities and others by developing more effective sign language recognition systems. | To enhance communication and interaction for the deaf and speech-impaired individuals. |
| N°78  Xiang (2023), China [78] | To construct prediction models of disability risk for geriatric patients with hypertension at different time intervals, as well as to assess the important predictors and influencing factors of disability. | Prediction | Mobility | small sample size | Not addressed | Sample diversity | To hold promise for screening out hypertensive old adults at high risk of disability. |
| N° 79  Yadav (2024), Spain [79] | To develop an AI-based approach to learning the relationship between the set of questionnaires and estimating the corresponding QoL dimensions. | Prediction | Cognition | Not addressed | Not addressed | Sample diversity | To pioneer DL-based approach for predicting the value of QoL dimensions |
| N° 80  Yi (2023), China  [80] | To analyse disease progression in Alzheimer's disease (AD) to identify distinct patterns in the trajectory from cognitively normal to mild cognitive impairment to AD dementia. | Prediction | Cognition | Missing data, unstable training of machine learning models, and neglect of individualized differences in time-to-conversion in existing models. | Not addressed | No | To help early prognosis of dementia, facilitate clinical trial design, and potentially aid in decision-making for clinicians. |
| N°81  Zarchi (2018), Iran [81] | To develop a database for self-care problems classification of children with physical and motor disability. | Prediction | Mobility | Not mentioned | Not addressed | Sample diversity | To highlight high applicability to be employed in the different classification systems such as neural networks and rule-based systems. |
| N°82  Zhang (2018), USA [82] | To improve dementia diagnosis using a quantitative approach to address the issue of heterogeneity in the diagnostic process. | Prediction | Cognition | Time and memory complexity | Not addressed | Sample diversity | To highlight the use of Framework that can be applied to other diseases with clinical heterogeneity, such as Parkinson’s disease. |
| N°83  Zhang (2022), China [83] | To develop and validate a deep learning model for screening fetuses with trisomy 21 based on ultrasonographic images. | Prediction | Cognition | Small sample size | Not addressed | Sample diversity | To facilitate universal primary screening for trisomy 21 |
| N°84  Zheng (2022), China [84] | The feasibility of using hybrid convolutional neural networks and long-short-term memory (CNN-LSTM) modeling for early decision-tailoring based on predicted long-term cognitive conversion in Alzheimer's disease (AD) and mild cognitive impairment (MCI). | Prediction | Cognition | Poor compliance to advanced neuroimaging techniques and difficulties in collecting corresponding information in real clinical practice. | Not addressed | No | To integrating AI strategies into routine clinical practice for Alzheimer's disease. |
| N°85  Cabrera-León (2024), Spain  [85] | To develop and evaluate a hybrid neural computing system (MyGNG) for early diagnosis of Alzheimer's disease (AD) and mild cognitive impairment (MCI). | Classification and prediction of AD and MCI for early diagnosis | Cognition | Limited generalizability, lack of racial diversity, small sample size | Use of neuropsychological tests, low-cost features, modular architecture. | Yes | Scalable early screening for AD, integration into eHealth platforms, support for personalized care and clinical decision-making. |
| N°86  Chen (2023), China [86] | To develop a low-cost, non-invasive multi-modal AI method for early diagnosis of MCI and AD using EEG, eye tracking, and behavioural data. | Prediction and classification of AD and MCI; early diagnosis support. | Cognition | Small sample size, individual EEG variability, limited generalizability, data imbalance. | Multi-modal data fusion, task-specific paradigms, EEG channel selection, ERPNet module. | Yes | Potential for scalable dementia screening, improved early intervention, and reduced diagnostic burden in clinical settings. |
| N°87  Yew ( 2024), Spain  [87] | To predict developing multiple chronic conditions (MCC) among people with ADRD using machine learning adjusted by syndemic factors. | Prediction of future chronic conditions based on existing MCC and syndemic factors. | Cognition | Imbalanced data, limited predictors for non-ADRD conditions, lack of racial diversity | Syndemic factors (social, mental, physical health, dementia severity) improved prediction accuracy. | Yes | Early identification of MCC in ADRD patients, improved clinical decision-making, potential for personalized care and reduced diagnostic burden. |
| N°88  Zhou (2025), China  [88] | To develop deep learning models using wearable IMU data for precise scoring of Fugl-Meyer Assessment (FMA) and prediction of Brunnstrom stages in post-stroke patients. | Prediction and scoring of motor function (FMA), classification of Brunnstrom stages. | Cognition | Wearable IMU sensors, GRU networks, PCA for dimensionality reduction, and clinical trial data. | Data imbalance, lack of racial diversity, and absence of an integrated software platform. | Yes | Enhancing precision rehabilitation, reducing clinician burden, enabling scalable home-based motor assessments, and informing future development of integrated platforms. |
| N°89  Ding ( 2025), China [89] | To predict Parkinson's disease (PD) | Prediction | Mental | Not mentioned | Data limitation | Yes | Optimize model parameters and enhance efficiency for prediction. |
| N°90  Adelson (2024), USA [90] | To predict mild cognitive impairment (MCI) | Prediction | Mental | Not mentioned | Data limitation | No | Facilitate care coordination, decrease healthcare expenditures, and maintain quality of life for patients at risk of progressing from MCI to AD. |
| N°91  Farooq (2025), China [91] | Early detection of Autism Spectrum Disorder (ASD) | Early detection | Mental | Facilities theparticipation of children | Low sample size | No | Development of neurological features in young children with ASD. |
| N°  Hatami (2024), Iran [92] | To develop an intelligent and accurate method for AD detection. | Detection | Mental | Not mentioned | Not mentioned | Yes | Proposing a datatype-independent framework based on RL to enhance the performance. |
| N°93  van Gils, A. M. (2024), Denmark [93] | To predict Dementia from f MRI-based brain volumetry | Prediction | Mental | Not mentioned | Not mentioned | No | To improve the classification accuracy and the discrimination of patients with Dementia. |

1.	Abbott, K., Y.-Y. Ho, and J. Erickson, *Automatic health record review to help prioritize gravely ill Social Security disability applicants.* Journal of the American Medical Informatics Association, 2017. **24**(4): p. 709-716.

2.	Akin-Bulbul, I., I. Kok, and S. Ozdemir, *Development of a visual attention based decision support system for autism spectrum disorder screening.* International Journal of Psychophysiology, 2022. **173**((Ozdemir) Hacettepe University, Hacettepe Education Faculty, Department of Special Education, Beytepe, Ankara, Turkey(Akin-Bulbul) Gazi University, Gazi Education Faculty, Special Education Department Teknikokullar, Ankara, Turkey(Kok) Pamukkale Universit): p. 69-81.

3.	Akter, S., et al., *AD-CovNet: An exploratory analysis using a hybrid deep learning model to handle data imbalance, predict fatality, and risk factors in Alzheimer's patients with COVID-19.* Computers in Biology and Medicine, 2022. **146**((Akter) Department of Bioinformatics and Computational Biology, George Mason University, Fairfax, VA, United States(Das) Department of Biochemistry and Molecular Biology, University of Dhaka, Dhaka, Bangladesh(Haque) School of Computer Science & Technolog): p. 105657.

4.	Almufareh, M.F., et al., *Artificial Cognition for Detection of Mental Disability: A Vision Transformer Approach for Alzheimer's Disease.* Healthcare (2227-9032), 2023. **11**(20): p. 2763.

5.	Alves, P., et al., *Validation of a machine learning approach to estimate expanded disability status scale scores for multiple sclerosis.* Multiple Sclerosis Journal - Experimental, Translational and Clinical, 2022. **8**(2).

6.	Ames, C.P., et al., *Artificial Intelligence Based Hierarchical Clustering of Patient Types and Intervention Categories in Adult Spinal Deformity Surgery: Towards a New Classification Scheme that Predicts Quality and Value.* Spine (03622436), 2019. **44**(13): p. 915-926.

7.	Assaf, M.H., et al., *Optimized tongue driven system using artificial intelligence.* Computer Methods in Biomechanics and Biomedical Engineering: Imaging and Visualization, 2023. **11**(3): p. 696-710.

8.	Baldo, F., et al., *Machine learning based analysis for intellectual disability in Down syndrome.* HELIYON, 2023. **9**(9).

9.	Bellemo, V., et al., *Artificial intelligence using deep learning to screen for referable and vision-threatening diabetic retinopathy in Africa: a clinical validation study.* The Lancet Digital Health, 2019. **1**(1): p. e35-e44.

10.	Ben-Shalom, Y. and D.C. Stapleton, *Predicting Disability among Community-Dwelling Medicare Beneficiaries Using Claims-Based Indicators.* Health services research, 2016. **51**(1): p. 262-81.

11.	Benabid, A.L., et al., *An exoskeleton controlled by an epidural wireless brain-machine interface in a tetraplegic patient: a proof-of-concept demonstration.* The Lancet. Neurology, 2019. **18**(12): p. 1112-1122.

12.	Berg, B., et al., *Machine Learning Models for Predicting Disability and Pain Following Lumbar Disc Herniation Surgery.* JAMA Network Open, 2024. **7**(2): p. e2355024-e2355024.

13.	Bertoncelli, C.M., et al., *PredictMed: A logistic regression–based model to predict health conditions in cerebral palsy.* Health Informatics Journal, 2020. **26**(3): p. 2105-2118.

14.	Bertoncelli, C.M., et al., *Predictive Model for Gastrostomy Placement in Adolescents With Developmental Disabilities and Cerebral Palsy.* Nutrition in Clinical Practice, 2020. **35**(1): p. 149-156.

15.	Bhagat, N.A., et al., *Neural activity modulations and motor recovery following brain-exoskeleton interface mediated stroke rehabilitation.* NeuroImage: Clinical, 2020. **28**((Bhagat, Hernandez, Contreras-Vidal) Non-Invasive Brain Machine Interface Systems Laboratory, University of Houston, Houston, TX 77004, United States(Yozbatiran, Paranjape, Keser, Francisco, O'Malley) Department of Physical Medicine and Rehabilitation, Mc): p. 102502.

16.	Bhagwat, N., et al., *An artificial neural network model for clinical score prediction in Alzheimer disease using structural neuroimaging measures.* Journal of psychiatry & neuroscience : JPN, 2019. **44**(4): p. 246-260.

17.	Bochniewicz, E.M., et al., *Measuring Functional Arm Movement after Stroke Using a Single Wrist-Worn Sensor and Machine Learning.* Journal of Stroke and Cerebrovascular Diseases, 2017. **26**(12): p. 2880-2887.

18.	Bragg, P.G., et al., *Application of supervised machine learning algorithms for the evaluation of utricular function on patients with Meniere's disease: utilizing subjective visual vertical and ocular-vestibular-evoked myogenic potentials.* Acta Oto-Laryngologica, 2023. **143**(4): p. 262-273.

19.	Brandsen, S., et al., *Prevalence of bias against neurodivergence-related terms in artificial intelligence language models.* AUTISM RESEARCH, 2024. **17**(2): p. 234-248.

20.	Briend, F., et al., *Voice acoustics allow classifying autism spectrum disorder with high accuracy.* Translational Psychiatry, 2023. **13**(1): p. 250.

21.	Bryan, M.J., L. Preston Jiang, and P.N.R. R, *Neural co-processors for restoring brain function: results from a cortical model of grasping.* Journal of Neural Engineering, 2023. **20**(3): p. 036004.

22.	Cantillo-Negrete, J., et al., *Brain-Computer Interface Coupled to a Robotic Hand Orthosis for Stroke Patients' Neurorehabilitation: A Crossover Feasibility Study.* Frontiers in human neuroscience, 2021. **15**(101477954): p. 656975.

23.	Cartwright, J., K. Kipp, and A.V. Ng, *Innovations in Multiple Sclerosis Care: The Impact of Artificial Intelligence via Machine Learning on Clinical Research and Decision-Making.* International Journal of MS Care, 2023. **25**(5): p. 233-241.

24.	Chen, S., et al., *A Multi-Modal Classification Method for Early Diagnosis of Mild Cognitive Impairment and Alzheimer's Disease Using Three Paradigms With Various Task Difficulties.* IEEE TRANSACTIONS ON NEURAL SYSTEMS AND REHABILITATION ENGINEERING, 2024. **32**: p. 1456-1465.

25.	Cheng, N., et al., *Brain-Computer Interface-Based Soft Robotic Glove Rehabilitation for Stroke.* IEEE transactions on bio-medical engineering, 2020. **67**(12): p. 3339-3351.

26.	Cho, K., J. Choi, and S. Han, *Validation of depression determinants in caregivers of dementia patients with machine learning algorithms and statistical model.* Frontiers in Medicine, 2023. **10**((Cho, Choi, Han) Yonsei Graduate Program in Cognitive Science, Yonsei University, Seoul, South Korea(Han) Department of Psychology, Yonsei University, Seoul, South Korea): p. 1095385.

27.	Connell, L.A., et al., *Implementing the PREP2 Algorithm to Predict Upper Limb Recovery Potential After Stroke in Clinical Practice: A Qualitative Study.* Physical therapy, 2021. **101**(5).

28.	Crowson, M.G., et al., *AutoAudio: Deep Learning for Automatic Audiogram Interpretation.* Journal of Medical Systems, 2020. **44**(9): p. N.PAG-N.PAG.

29.	de Vries, S., et al., *Real-time stress detection based on artificial intelligence for people with an intellectual disability.* ASSISTIVE TECHNOLOGY, 2023.

30.	Dosso, J.A., J.N. Kailley, and J.M. Robillard, *What Does ChatGPT Know About Dementia? A Comparative Analysis of Information Quality.* Journal of Alzheimer's Disease, 2024. **97**(2): p. 559-565.

31.	Farhadian, M., M. Aliabadi, and E. Darvishi, *Empirical estimation of the grades of hearing impairment among industrial workers based on new artificial neural networks and classical regression methods.* Indian Journal of Occupational and Environmental Medicine, 2015. **19**(2): p. 84-89.

32.	Frazier, T.W., et al., *Development of webcam-collected and artificial-intelligence-derived social and cognitive performance measures for neurodevelopmental genetic syndromes.* AMERICAN JOURNAL OF MEDICAL GENETICS PART C-SEMINARS IN MEDICAL GENETICS, 2023. **193**(3).

33.	Fromm, D., et al., *Enhancing the classification of aphasia: a statistical analysis using connected speech.* Aphasiology, 2022. **36**(12): p. 1492-1519.

34.	Gao, F., et al., *A combination model of AD biomarkers revealed by machine learning precisely predicts Alzheimer's dementia: China Aging and Neurodegenerative Initiative (CANDI) study.* Alzheimer's and Dementia, 2023. **19**(3): p. 749-760.

35.	Geng, C., Z. Wang, and Y. Tang, *Machine learning in Alzheimer's disease drug discovery and target identification.* Ageing Research Reviews, 2024. **93**((Geng, Wang, Tang) Department of Neurology & Innovation Center for Neurological Disorders, Xuanwu Hospital, Capital Medical University, National Center for Neurological Disorders, Beijing, China(Tang) Neurodegenerative Laboratory of Ministry of Education): p. 102172.

36.	Gharbi-Meliani, A., et al., *Identification of high likelihood of dementia in population-based surveys using unsupervised clustering: a longitudinal analysis.* Alzheimer's Research and Therapy, 2023. **15**(1): p. 209.

37.	Grassi, M., et al., *A novel ensemble-based machine learning algorithm to predict the conversion from mild cognitive impairment to Alzheimer's disease using socio-demographic characteristics, clinical information, and neuropsychological measures.* Frontiers in Neurology, 2019. **10**(JUL): p. 756.

38.	Guan, R., et al., *Trends in Alzheimer's Disease Research Based upon Machine Learning Analysis of PubMed Abstracts.* International journal of biological sciences, 2019. **15**(10): p. 2065-2074.

39.	Hart, K.L., et al., *Distribution of agitation and related symptoms among hospitalized patients using a scalable natural language processing method.* General Hospital Psychiatry, 2021. **68**: p. 46-51.

40.	He, L., et al., *Deep Multimodal Learning From MRI and Clinical Data for Early Prediction of Neurodevelopmental Deficits in Very Preterm Infants.* Frontiers in Neuroscience, 2021. **15**((He, Li, Chen, Dillman) Imaging Research Center, Cincinnati Children's Hospital Medical Center, Cincinnati, OH, United States(He, Li, Chen, Dillman) Department of Radiology, Cincinnati Children's Hospital Medical Center, Cincinnati, OH, United States(He): p. 753033.

41.	Hill, A., D.R. Mann, and J. Gellar, *Predicting program outcomes for vocational rehabilitation customers: A machine learning approach.* Journal of Vocational Rehabilitation, 2022. **56**(2): p. 107-121.

42.	Hruby, L.A., et al., *Algorithm for bionic hand reconstruction in patients with global brachial plexopathies.* Journal of neurosurgery, 2017. **127**(5): p. 1163-1171.

43.	Hu, M.Y. and J.Y. Wang, *Artificial intelligence in dance education: Dance for students with special educational needs.* TECHNOLOGY IN SOCIETY, 2021. **67**.

44.	Imbiriba, T., et al., *Wearable Biosensing to Predict Imminent Aggressive Behavior in Psychiatric Inpatient Youths with Autism.* JAMA Network Open, 2023. **6**(12): p. E2348898.

45.	Ingelse, L., et al., *Personalised Gait Recognition for People with Neurological Conditions.* Sensors (Basel, Switzerland), 2022. **22**(11).

46.	Janssen, E.R., et al., *Exploring Associations of Preoperative Physical Performance With Postoperative Outcomes After Lumbar Spinal Fusion: A Machine Learning Approach.* Archives of physical medicine and rehabilitation, 2021. **102**(7): p. 1324-1330.e3.

47.	Justice, L.M., W.-Y. Ahn, and J.A.R. Logan, *Identifying Children With Clinical Language Disorder: An Application of Machine-Learning Classification.* Journal of learning disabilities, 2019. **52**(5): p. 351-365.

48.	Kang, K., et al., *Bayesian adaptive group lasso with semiparametric hidden Markov models.* Statistics in Medicine, 2019. **38**(9): p. 1634-1650.

49.	Kang, W., et al., *Multi-model and multi-slice ensemble learning architecture based on 2D convolutional neural networks for Alzheimer's disease diagnosis.* Computers in biology and medicine, 2021. **136**(doc, 1250250): p. 104678.

50.	Kanzler, C.M., et al., *Personalized prediction of rehabilitation outcomes in multiple sclerosis: a proof-of-concept using clinical data, digital health metrics, and machine learning.* Medical & biological engineering & computing, 2022. **60**(1): p. 249-261.

51.	Kim, S.Y., et al., *Diagnostic validity of Autism Diagnostic Observation Schedule, second edition (K-ADOS-2) in the Korean population.* Molecular Autism, 2022. **13**(1): p. 30.

52.	Klein, K.E., et al., *Using a Digital Language Processor to Quantify the Auditory Environment and the Effect of Hearing Aids for Adults with Hearing Loss.* Journal of the American Academy of Audiology, 2018. **29**(4): p. 279-291.

53.	Lee, S., et al., *Effects of altered functional connectivity on motor imagery brain-computer interfaces based on the laterality of paralysis in hemiplegia patients.* COMPUTERS IN BIOLOGY AND MEDICINE, 2023. **166**.

54.	Lindsay, H., J. Troger, and A. Konig, *Language Impairment in Alzheimer's Disease-Robust and Explainable Evidence for AD-Related Deterioration of Spontaneous Speech Through Multilingual Machine Learning.* Frontiers in aging neuroscience, 2021. **13**(101525824): p. 642033.

55.	Lombardi, M., et al., *Factors predicting quality of life for people with intellectual disability: Results from the ANFFAS study in Italy.* Journal of Intellectual & Developmental Disability, 2016. **41**(4): p. 338-347.

56.	Mahmut, E.-E., S. Nicola, and V. Stoicu-Tivadar, *Support-Vector Machine-Based Classifier of Cross-Correlated Phoneme Segments for Speech Sound Disorder Screening.* Studies in health technology and informatics, 2022. **294**(ck1, 9214582): p. 455-459.

57.	Marrie, R.A., et al., *Development and Internal Validation of a Disability Algorithm for Multiple Sclerosis in Administrative Data.* Frontiers in Neurology, 2021. **12**((Marrie, Marriott) Department of Internal Medicine, Max Rady College of Medicine, Rady Faculty of Health Sciences, University of Manitoba, Winnipeg, MB, Canada(Marrie) Department of Community Health Sciences, Max Rady College of Medicine, Rady Faculty of): p. 754144.

58.	McCombe, N., et al., *Multiple Cost Optimisation for Alzheimer's Disease Diagnosis.* medRxiv, 2022((McCombe, Ding, Prasad, Wong-Lin) Intelligent Systems Research Centre, Ulster University, Magee Campus, Derry, Londonderry, United Kingdom(Finn) Pharmacology and Therapeutics, School of Medicine, Galway Neuroscience Centre, Centre for Pain Research, Natio).

59.	McCombe, N., et al., *Alzheimer's Disease Assessments Optimised for Diagnostic Accuracy and Administration Time.* medRxiv, 2021((McCombe, Ding, Prasad, Wong-Lin) Intelligent Systems Research Centre, Ulster University, Magee campus, Derry~Londonderry, Northern Ireland (NI), United Kingdom(Gillespie) Health Economic and Policy Analysis Centre, Discipline of Economics, National Unive).

60.	Meskers, C.G.M., et al., *Automated recognition of functioning, activity and participation in COVID-19 from electronic patient records by natural language processing: a proof- of- concept.* Annals of Medicine, 2022. **54**(1): p. 235-243.

61.	Moreno Escobar, J.J., et al., *DS-CNN: Deep Convolutional Neural Networks for Facial Emotion Detection in Children with Down Syndrome during Dolphin-Assisted Therapy.* Healthcare (2227-9032), 2023. **11**(16): p. 2295.

62.	Nikam, V., et al., *A pilot study on machine learning approach to delineate metabolic signatures in intellectual disability.* International Journal of Developmental Disabilities, 2021. **67**(2): p. 94-100.

63.	Noel, J.P., et al., *Inflexible Updating of the Self-Other Divide During a Social Context in Autism: Psychophysical, Electrophysiological, and Neural Network Modeling Evidence.* Biological Psychiatry: Cognitive Neuroscience and Neuroimaging, 2022. **7**(8): p. 756-764.

64.	Ogawa, H., et al., *Can Deep Generative Models Explain Brain Function in People with Developmental Dyslexia?* ELECTRONICS, 2023. **12**(10).

65.	Olivares-Gil, A., et al., *A low-cost system using a big-data deep-learning framework for assessing physical tele-rehabilitation: A proof-of-concept.* Movement Disorders, 2022. **37**(Supplement 2): p. S461.

66.	Oscar, N., et al., *Machine Learning, Sentiment Analysis, and Tweets: An Examination of Alzheimer's Disease Stigma on Twitter.* Journals of Gerontology Series B: Psychological Sciences & Social Sciences, 2017. **72**(5): p. 742-751.

67.	Panganiban, J. and C. Kasari, *Super responders: Predicting language gains from JASPER among limited language children with autism spectrum disorder.* Autism Research, 2022. **15**(8): p. 1565-1575.

68.	Schilling, A., et al., *Predictive coding and stochastic resonance as fundamental principles of auditory phantom perception.* Brain, 2023. **146**(12): p. 4809-4825.

69.	Shehzad, A., et al., *Use of Patient-Reported Symptoms from an Online Symptom Tracking Tool for Dementia Severity Staging: Development and Validation of a Machine Learning Approach.* Journal of medical Internet research, 2020. **22**(11): p. e20840.

70.	Steinheimer, S., et al., *Setwise comparison: efficient fine-grained rating of movement videos using algorithmic support - a proof of concept study.* Disability and rehabilitation, 2020. **42**(18): p. 2640-2646.

71.	Tham, Y.-C., et al., *Referral for disease-related visual impairment using retinal photograph-based deep learning: a proof-of-concept, model development study.* The Lancet. Digital health, 2021. **3**(1): p. e29-e40.

72.	Tuechler, K., et al., *Mapping patient reported outcome measures for low back pain to the International Classification of Functioning, Disability and Health using random forests.* European journal of physical and rehabilitation medicine, 2020. **56**(3): p. 286-296.

73.	Tunthanathip, T. and T. Oearsakul, *Application of machine learning to predict the outcome of pediatric traumatic brain injury.* Chinese Journal of Traumatology, 2021. **24**(6): p. 350-355.

74.	Valcarcel, A.M., et al., *A dual modeling approach to automatic segmentation of cerebral T2 hyperintensities and T1 black holes in multiple sclerosis.* NeuroImage: Clinical, 2018. **20**((Valcarcel, Linn, Vandekar, Martin, Shinohara) Department of Biostatistics, Epidemiology, and Informatics, Perelman School of Medicine, University of Pennsylvania, Philadelphia, PA, United States(Khalid, Tauhid, Bakshi) Laboratory for Neuroimaging Researc): p. 1211-1221.

75.	Vaucheret Paz, E., et al., *Sentiment Analysis in Children with Neurodevelopmental Disorders in an Ingroup/Outgroup Setting.* Journal of Autism and Developmental Disorders, 2020. **50**(1): p. 162-170.

76.	Verma, D., et al., *Exploratory application of machine learning methods on patient reported data in the development of supervised models for predicting outcomes.* BMC medical informatics and decision making, 2022. **22**(1): p. 227.

77.	Wategaonkar, D., et al., *SIGN GESTURE INTERPRETER FOR BETTER COMMUNICATION BETWEEN A NORMAL AND DEAF PERSON.* Journal of Pharmaceutical Negative Results, 2022. **13**((Wategaonkar, Pawar, Jadhav, Patole, Jadhav, Gupta) School of Computer Engineering and Technology, Dr. Vishwanath Karad MIT World Peace University, Pune, India): p. 5990-6000.

78.	Xiang, C., et al., *Machine learning-based prediction of disability risk in geriatric patients with hypertension for different time intervals.* Archives of Gerontology & Geriatrics, 2023. **105**: p. N.PAG-N.PAG.

79.	Yadav, G.K., et al., *A Data-Driven Model to Predict Quality of Life Dimensions of People with Intellectual Disability Based on the GENCAT Scale.* SOCIAL INDICATORS RESEARCH, 2024.

80.	Yi, F., et al., *Identifying underlying patterns in Alzheimer's disease trajectory: a deep learning approach and Mendelian randomization analysis.* eClinicalMedicine, 2023. **64**((Yi, Hao, Wu, Huang) College of Computer Science and Technology, Zhejiang University, China(Zhang) DAMO Academy, Alibaba Group, China(Yuan, Liu, Zhai, Zhu) Department of Neurology, Peking Union Medical College Hospital, Peking Union Medical College, Chine): p. 102247.

81.	Zarchi, M.S., S.M.M. Fatemi Bushehri, and M. Dehghanizadeh, *SCADI: A standard dataset for self-care problems classification of children with physical and motor disability.* International Journal of Medical Informatics, 2018. **114**: p. 81-87.

82.	Zhang, H., et al., *A similarity-based approach to leverage multi-cohort medical data on the diagnosis and prognosis of Alzheimer's disease.* GigaScience, 2018. **7**(7): p. giy085.

83.	Zhang, L., et al., *Development and Validation of a Deep Learning Model to Screen for Trisomy 21 During the First Trimester From Nuchal Ultrasonographic Images.* JAMA Network Open, 2022. **5**(6): p. e2217854-e2217854.

84.	Zheng, Y., et al., *Predicted Cognitive Conversion in Guiding Early Decision-Tailoring on Patients With Cognitive Impairment.* Frontiers in Aging Neuroscience, 2022. **13**((Zheng) Department of Rehabilitation Medicine, The First Affiliated Hospital of Nanjing Medical University, Nanjing, China(Liu, Wu, Yang, Li, Sun, Wu) Division of Brain Rehabilitation, Department of Neurology, The First Affiliated Hospital of Nanjing Medi): p. 813923.

85.	Cabrera-León, Y., et al., *Toward an intelligent computing system for the early diagnosis of Alzheimer’s disease based on the modular hybrid growing neural gas.* Digital Health, 2024. **10**: p. 20552076241284349.

86.	Chen, Y.-W., et al., *Predicting patient-reported outcome of activities of daily living in stroke rehabilitation: a machine learning study.* Journal of neuroengineering and rehabilitation, 2023. **20**(1): p. 25.

87.	Yew, P.Y., et al., *Unraveling the multiple chronic conditions patterns among people with Alzheimer's disease and related dementia: A machine learning approach to incorporate synergistic interactions.* Alzheimer's and Dementia, 2024. **20**(7): p. 4818EP-4827.

88.	Zhou, W.N., et al., *Achieving precision assessment of functional clinical scores for upper extremity using IMU-Based wearable devices and deep learning methods.* JOURNAL OF NEUROENGINEERING AND REHABILITATION, 2025. **22**(1).

89.	Ding, Q.Y., et al., *AMEF-Net: Towards an attention and multi-level enhancement fusion for medical image classification in Parkinson's aided diagnosis.* IET COMPUTER VISION, 2025. **19**(1).

90.	Adelson, R.P., et al., *Machine Learning Approach for Improved Longitudinal Prediction of Progression from Mild Cognitive Impairment to Alzheimer's Disease.* DIAGNOSTICS, 2024. **14**(1).

91.	Farooq, S., et al., *Advanced autism detection and visualization through XGBoost algorithm for fNIRS hemo-dynamic signals.* EXPERT SYSTEMS WITH APPLICATIONS, 2025. **275**.

92.	Hatami, M., F. Yaghmaee, and R. Ebrahimpour, *Investigating the potential of reinforcement learning and deep learning in improving Alzheimer's disease classification.* Neurocomputing, 2024. **597**((Hatami, Yaghmaee) Department of Electrical & Computer Engineering, Semnan University, Semnan, Iran, Islamic Republic of(Ebrahimpour) Center for Cognitive Science, Institute for Convergence Science and Technology, Sharif University of Technology, Tehran): p. 128119.

93.	van Gils, A.M., et al., *Separating dementia with Lewy bodies from Alzheimer's disease dementia using a volumetric MRI classifier.* EUROPEAN RADIOLOGY, 2024.
